# Bacteria and Virus Inactivation: Relative Efficacy and Mechanisms of Peroxyacids and Chlor(am)ine

**DOI:** 10.1021/acs.est.2c09824

**Published:** 2023-03-30

**Authors:** Junyue Wang, Wensi Chen, Ting Wang, Elliot Reid, Caroline Krall, Juhee Kim, Tianqi Zhang, Xing Xie, Ching-Hua Huang

**Affiliations:** † School of Civil and Environmental Engineering, Georgia Institute of Technology, Atlanta, Georgia 30332, United States; ‡ School of Architecture, Civil and Environmental Engineering (ENAC), École Polytechnique FÉdÉrale de Lausanne (EPFL), 1015 Lausanne, Switzerland

**Keywords:** peroxyacids (POAs), bacterial inactivation, viral inactivation, wastewater disinfection, fluorescence microscopy

## Abstract

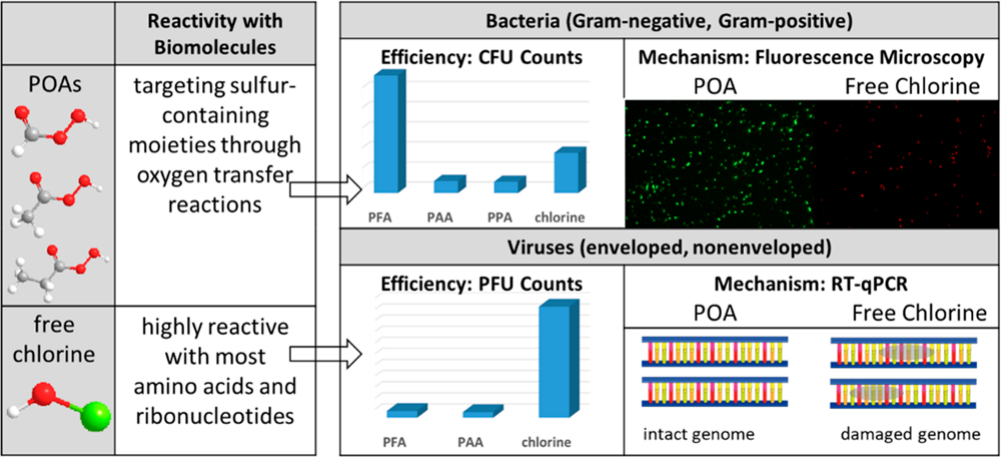

Peroxyacids (POAs) are a promising alternative to chlorine for reducing the formation of disinfection byproducts. However, their capacity for microbial inactivation and mechanisms of action require further investigation. We evaluated the efficacy of three POAs (performic acid (PFA), peracetic acid (PAA), and perpropionic acid (PPA)) and chlor(am)ine for inactivation of four representative microorganisms (*Escherichia coli* (Gram-negative bacteria), *Staphylococcus epidermidis* (Gram-positive bacteria), MS2 bacteriophage (nonenveloped virus), and Φ6 (enveloped virus)) and for reaction rates with biomolecules (amino acids and nucleotides). Bacterial inactivation efficacy (in anaerobic membrane bioreactor (AnMBR) effluent) followed the order of PFA > chlorine > PAA ≈ PPA. Fluorescence microscopic analysis indicated that free chlorine induced surface damage and cell lysis rapidly, whereas POAs led to intracellular oxidative stress through penetrating the intact cell membrane. However, POAs (50 μM) were less effective than chlorine at inactivating viruses, achieving only ∼1-log PFU removal for MS2 and Φ6 after 30 min of reaction in phosphate buffer without genome damage. Results suggest that POAs’ unique interaction with bacteria and ineffective viral inactivation could be attributed to their selectivity toward cysteine and methionine through oxygen-transfer reactions and limited reactivity for other biomolecules. These mechanistic insights could inform the application of POAs in water and wastewater treatment.

## Abstract 2

This paper systematically compared POAs and chlorine for bacterial and viral inactivation, and studied the mechanisms by fluorescence microscopy, RT-qPCR, and mass spectrometry.

## 1 Introduction

Disinfection of water and wastewater has played an essential role in improving public health over the last few decades.^1−4^ Disinfectants are primarily strong oxidants that efficiently inactivate pathogens and protect populations from waterborne diseases, including diarrhea, cholera, salmonellosis, giardiasis, and cryptosporidiosis. Chlorine-based disinfectants, including free chlorine (HOCl/ClO^–^) and combined chlorine (NH_2_Cl, NHCl_2_), are the most commonly used disinfectants due to their relatively low costs and high inactivation effectiveness.^2,3^ However, the formation of halogenated disinfection byproducts (DBPs) during chlor(am)ination warrants public concerns. To alleviate the DBP problems during disinfection, peroxyacids (POAs, R–C(O)OOH), a group of carboxylic acid–based peroxides, are proposed as a replacement to chlorine-based disinfectants for wastewater disinfection.^5^ While POAs provide effective avoidance of halogenated DBPs,^6,7^ the pathogen inactivation capacity and mechanisms of POAs are not yet well-understood and require comprehensive investigation and systematic comparison with chlorine.

For bacterial inactivation, peracetic acid (PAA, H_3_C–C(O)OOH) has been widely investigated due to its chemical stability and commercialized products on the market. PAA is reported to have comparable disinfection efficiency with free chlorine in wastewater effluents and has been applied in many wastewater treatment plants.^8,9^ More recently, performic acid (PFA, H–C(O)OOH) has emerged as another promising POA for water disinfection, and studies have reported PFA outperforming PAA for inactivation of *Escherichia coli* and *Enterococci*.^9−16^ Meanwhile, perpropionic acid (PPA, H_5_C_2_–C(O)OOH), another POA with a longer alkyl chain than PAA and PFA, may exhibit similar chemical properties but has been scarcely studied for pathogen inactivation.

Previous studies mostly compared the disinfection of POAs and chlorine in wastewater effluents from activated sludge processes.^9−16^ Compared to aerobic processes, anaerobic treatment avoids the energy-intensive aeration and produces biogas (e.g., methane, hydrogen) that could be used for electricity/heat cogeneration.^17^ In particular, an anaerobic membrane bioreactor (AnMBR), taking advantages of the superior micropollutant removal and prolonged solid retention time (SRT) by ultrafiltration, has been widely investigated in the past decades.^18^ The effluents from anaerobic processes are distinctly different from the aerobic effluents, particularly in ammonia concentration, which may affect disinfection efficiency. As reported, the average ammonia concentration from anaerobic treatment is as high as 36 ± 17 mg-N·L^–1^ due to the lack of aerobic nitrification units.^17^ To our knowledge, the disinfection of effluents from anaerobic bioreactors by POAs has never been investigated until this study.

Moreover, the interaction mechanisms between POAs and bacteria have not been compared with those of chlorine. It has been reported that strong oxidants with low selectivity (e.g., free chlorine^19−22^ and ozone^20,22,23^) damage the cell surfaces and induce cell lysis, which in turn gives rise to release of intracellular polymeric substances (IPS) (serving as important DBP precursors and membrane foulants) and intracellular antibiotic resistance genes (iARGs).^24−26^ Dukan et al. reported that free chlorine was easily consumed by extracellular polymeric substances (EPS) or IPS and, hence, was more difficult to accumulate inside the cell and cause intracellular damage.^27^ In contrast, selective oxidants like PAA, with low reactivity with cell membrane and EPS, may diffuse through intact membrane and become accumulated intracellularly, while leaving the bacteria in viable but nonculturable states (VBNC).^27−29^ To date, a systematic comparison between POAs and chlorine has not been demonstrated at a cellular level to evaluate their inactivation mechanisms.

For inactivation of viruses, significant research has been conducted to study PAA’s ability to inactivate nonenveloped virus surrogates. From the literature, PAA can inactivate human norovirus surrogates (e.g., Murine norovirus (MNV),^15,30,31^ Feline calicivirus,^30^ Tulane virus^32^) and rotavirus^32^ to a satisfactory level, but the removal of Hepatitis A,^30^ bacteriophage P001,^33^ and MS2^31,33^ is inefficient. On the other hand, viral inactivation by PFA has been scarcely reported and whether PFA can inactivate the PAA-resistant viruses is worthy of research. Furthermore, to our knowledge, the disinfection effectiveness toward enveloped viruses has not been studied for PAA or PFA. Since enveloped viruses are generally more vulnerable to oxidation than nonenveloped viruses,^34−37^ previous research has hypothesized that POAs could inactivate enveloped viruses more efficiently.^35^ However, the effectiveness of POAs on enveloped viruses has never been experimentally confirmed.

Additionally, the viral inactivation mechanisms, i.e., protein and/or genome damage, of POAs require further investigation. As reported, free chlorine could induce protein damage (e.g., echovirus^38^) or nonspecific damage on proteins/genomes (e.g., MS2^39^ and Φ6^36^), whereas monochloramine mainly attacks the surface of pathogens with limited genome damage (e.g., adenovirus^40^). UV at 254 nm mainly triggers direct genome damage through dimerization of adjacent pyrimidine bases without significant damage of the proteins (e.g., adenovirus,^41^ echovirus,^38^ Φ6^36^). Ferrate(VI) and ozone are strong oxidants and give rise to both genome and protein damage.^38,42,43^ However, the viral inactivation mechanisms of PAA have been controversial. Fuzawa et al. reported that PAA damaged the genomes of Tulane virus and rotavirus,^44^ while Schmitz et al. concluded that PAA could not react with the MS2 genome.^45^ Furthermore, the viral inactivation mechanisms of PFA have never been investigated.

Therefore, to fill the above identified knowledge gaps on POA disinfection efficacy and mechanisms, we systematically compared the disinfection efficiency of three POAs (PFA, PAA, and PPA) and chlorine, with four representative surrogates, i.e., *Escherichia coli* (*E. coli*, Gram-negative bacteria), *Staphylococcus epidermidis* (*S. epidermidis*, Gram-positive bacteria), MS2 bacteriophage (nonenveloped virus), and Φ6 (enveloped virus). Complementary to disinfection experiments, fluorescence microscopy and reverse transcription-quantitative polymerase chain reaction (RT-qPCR) were employed to delineate the interaction mechanisms of the oxidants with bacteria and viruses, respectively. The reactivity of POAs with biomolecules (amino acids and nucleotides) was also evaluated to provide fundamental chemical understanding of POA disinfection.

## 2 Materials and Methods

### 2.1 Chemicals, Reagents, and Microbial Cultures

Due to its typical synthesis, PAA solution is a mixture of PAA, H_2_O_2_, acetic acid, and water.^46^ PAA solution (32% PAA and 6% H_2_O_2_ w/w in acetic acid and water solution) and hydrogen peroxide solution (30% H_2_O_2_ w/w in water) were purchased from Sigma-Aldrich (St. Louis, MO) and kept at 5 °C, and the oxidant concentrations were determined by iodometric and permanganate titration methods as described previously.^47,48^ For this study, additional H_2_O_2_ (20% of PAA, M/M) was dosed to the PAA solution in order to keep the H_2_O_2_ concentration in three POA solutions at similar levels (molar ratio of POA/H_2_O_2_ = 100:57-63). Free chlorine (NaOCl) solution (4.99% weight in water) was purchased from Sigma-Aldrich (St. Louis, MO). Sources of other chemicals, bacteria and virus cultures are described in Text S1.

PFA, PPA, and monochloramine were synthesized in our lab. Monochloramine was produced by mixing NaOCl with ammonia at a 1:1.2 molar ratio and adjusting the resultant solution to pH 8.5.^49^ PFA and PPA were synthesized from oxidation of formic acid and propionic acid, respectively. Details of the synthesis methods and yields for PFA and PPA are provided in Text S2 and Table S1.

### 2.2 Batch Disinfection Experiments

As bacterial inactivation by POAs has been compared with chlorine in DI water and aerobic wastewater effluents in previous studies,^9,10,14,15^ we employed effluents from a bench-scale anaerobic membrane bioreactor (AnMBR), which was seeded with anaerobic digestor sludge and maintained with synthetic wastewater feed (Text S3) as the water matrix for bacterial inactivation experiments. The characteristics of the synthetic wastewater feed and the AnMBR effluent are provided in Tables S2 and S3. The bacterial surrogate was spiked into phosphate-buffered AnMBR effluent in a 20 mL quartz reactor. Then, POA or free chlorine (NaOCl) was dosed to initiate the disinfection experiments. The solution was magnetically stirred and open to the ambient air, and pH was measured after POA addition and throughout the reaction. Periodically, 1 mL samples were taken from the reactor and immediately quenched by excess Na_2_S_2_O_3_. Then, the bacteria samples were serially diluted and plated on agars as described in Text S4. Separately, a regrowth test was conducted by incubating the inactivated bacteria samples (1 mL, oxidants quenched by excessive Na_2_S_2_O_3_) with 20 mL of corresponding nutrient broth at room temperature and measuring the absorbance at 600 nm at defined time intervals.^29^

Viral inactivation experiments were conducted in a similar way as described above, except that the disinfection was performed in clean phosphate buffer solutions instead of AnMBR effluents, due to the difficulties of virus culturing in wastewater containing various bacteria and the lack of a fundamental understanding for viral inactivation in clean water matrix thus far. Virus concentrations were determined by a double layer agar method (Text S4).^50^

### 2.3 Fluorescence Microscopy

To understand the bacterial inactivation mechanisms, 2,7-dichlorodihydrofluorescein diacetate (DCFH-DA) and propidium iodide (PI) were used as fluorophores to test intracellular oxidative stress and membrane integrity, respectively.^15^ For the DCFH-DA experiments, *E. coli* was incubated with 340 μM of DCFH-DA (pH = 7.1, phosphate buffer) for 20 min at 35 °C; then, one of the oxidants was added and the bacteria was observed under an inverted fluorescence microscope (Zeiss Axio observer 7) equipped with a charge-coupled device camera. For the PI experiment, *E. coli* treated by the oxidants (buffered at pH 7.1) for defined time intervals were collected and quenched by Na_2_S_2_O_3_. Then, 15 μM PI was added and the images were taken under the fluorescence microscope. Emission of DCF and PI was observed with a 63× water immersion lens at the excitation wavelengths of 488 and 555 nm, together with an FS 90 HE filter and FS 91 HE filter, respectively. Note that bacteria positions were consistent throughout each DCFH-DA experiment but not the PI experiments, where different samples were prepared for different reaction times. However, the bacteria concentrations remained the same in all the images for a fair comparison. The cell numbers in the images were counted using MATLAB, and the percentage of fluorescent cells was calculated and reported.

### 2.4 Genome Degradation Experiments

To evaluate the viral inactivation mechanisms, viral genome damage during disinfection by PFA, PAA, and free chlorine, was measured by RT-qPCR following previous methods (Text S5).^50,51^ The rate constant for genome damage was calculated (eqs 1 and 2), and the reaction of the whole genome was predicted through the extrapolation of the RT-qPCR results (eq 2).^36^
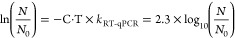
1
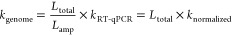
2where *k*_RT-qPCR_ and *k*_genome_ are the apparent degradation reaction rate constants of the RT-qPCR target regions and the entire genomes of the viruses, respectively, in L·mg^–1^·s^–1^; *L*_amp_ is the size of the qPCR amplicons, i.e., 83 bases for MS2 and 280 bases (140 base pairs) for Φ6 in this study; *L*_total_ is the size of the entire genome, i.e., 3600 bases for MS2 and 26 800 bases (13 400 base pairs) for Φ6; *k*_normalized_ is the normalized rate constant in L·mg^–1^·s^–1^·base^–1^; C·T is the cumulative exposure to oxidants (in mg·s·L^–1^); *N*_0_ is the initial concentration of genome (copies·mL^–1^); and *N* is the genome concentration after certain exposure to oxidants (copies·mL^–1^).

To test the functionality loss of the spike proteins of Φ6, the virus’ ability to recognize and attach to its host bacterium *P. syringae* was studied by the ice-bath^39^ or chloramphenicol^52^ methods (Text S6).

### 2.5 Analytical Methods

The concentrations of POAs were measured by the potassium iodide-*N*,*N*-diethyl-*p*-phenylenediamine (KI-DPD) method with a UV–visible spectrophotometer (Text S7).^53^ The total chlorine was determined by the DPD method.^49,54^ The total concentration of POA and coexisting H_2_O_2_ was determined by a horseradish peroxidase-2,2′-azino-bis(3-ethylbenzothiazoline-6-sulfonic)acid (HRP-ABTS) method. The H_2_O_2_ concentration was obtained by subtracting the POA concentration from the total peroxide concentration. (Text S7, Figure S1).

## 3 Results and Discussion

### 3.1 Inactivation of Bacteria in AnMBR Effluent

Although bacterial inactivation by POAs has been studied in clean matrix and aerobic wastewater effluents,^9,10,14,15^ POAs’ performance in effluents from anaerobic treatment (e.g., AnMBR) has not been studied. Anaerobic effluents contain a higher NH_4_^+^-N level due to the lack of aerobic nitrification units, which may affect the disinfection performance of common oxidants.^17^ The degradation of POAs and total chlorine was first studied in the AnMBR effluent. PAA and PPA were stable for 15 min in the matrix, indicating their chemical stability and low reactivity with the effluent organic matter (EfOM) (Figure S2a,b). In contrast, PFA decayed at pseudo-first-order rate constants of 0.068 and 0.082 min^–1^ at pH 7.1 and 7.8, respectively (Figure S2c,d), which were comparable to the values reported by Maffettone et al. (0.028–0.085 min^–1^)^15^ and Ragazzo et al. (0.031 min^–1^).^9^ The major reactions of PFA self-decay include eqs 3 and 4.^55^
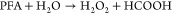
3
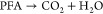
4

Contrary to PFA, total chlorine exhibited faster self-decay that could not be well-modeled by pseudo-first-order or second-order kinetics (Figure S2c,d), which was likely attributed to the instant chlorine demand by EfOM and speciation among free chlorine and inorganic/organic chloramines.^3,56^ With an ammonia concentration at 43.2 ± 2.6 mg·L^–1^ as N, all the free chlorine dosed into the reactors was ultimately converted to combined chlorine (monochloramine). However, as the free chlorine may inactivate bacteria before its reaction with ammonia, the resultant disinfection should be attributed to both free and combined chlorine. For easier discussion, we uniformly use the word “chlorine” to refer to the overall disinfection by free and/or combined chlorine even though free chlorine was the original form that was spiked into the reactors.

**Figure 1:**
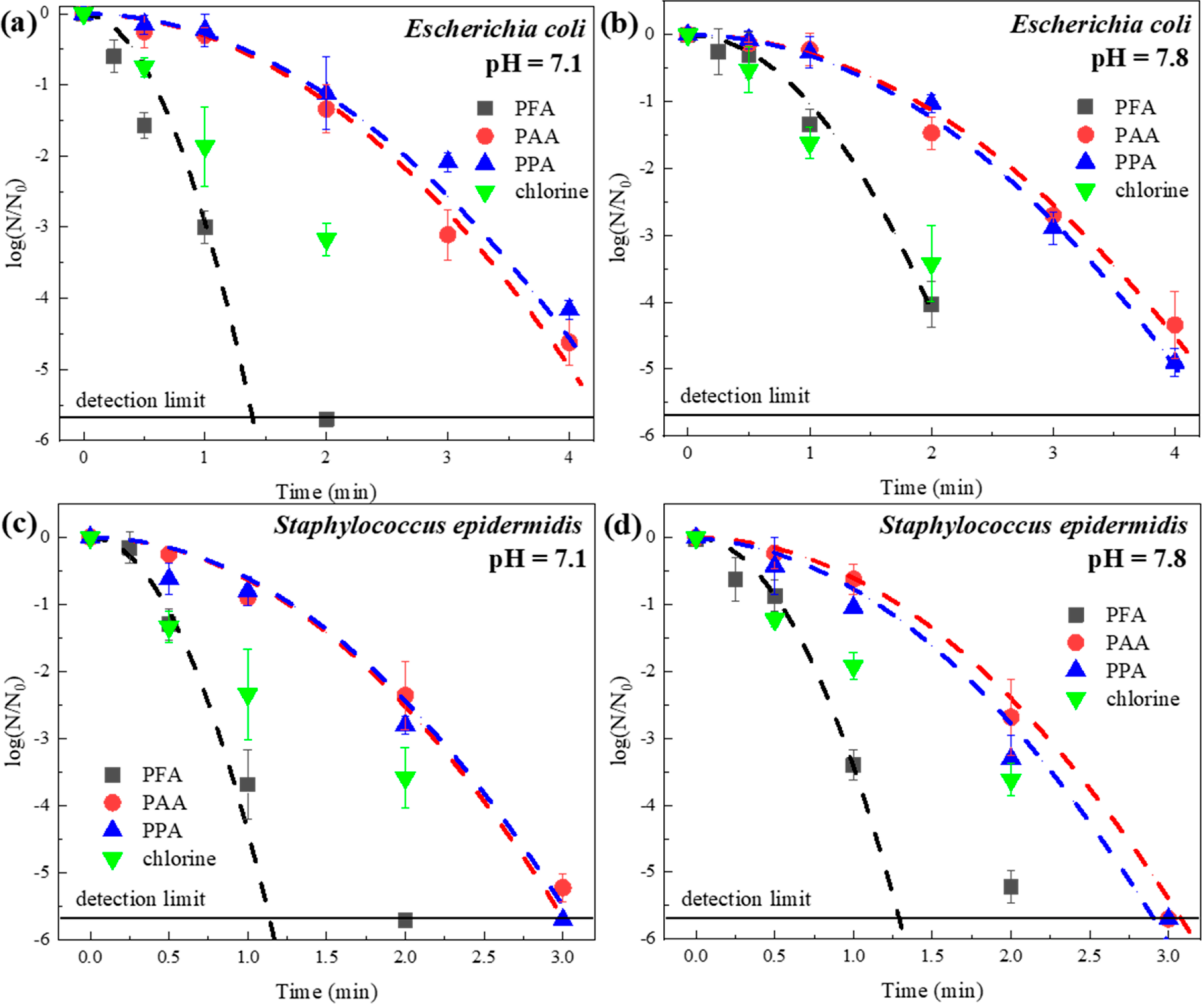
Inactivation of (a and b) *E. coli* and (c and d) *S. epidermidis* by POAs and chlorine in effluent from a bench-scale AnMBR. Experimental conditions: [POAs]_0_ = 120 μM (all with 68–75 μM coexistent H_2_O_2_), [total chlorine]_0_ = 120 μM, [cells]_0_ ≈ 1 × 10^7^ CFU·mL^–1^, [phosphate buffer] = 10 mM, temperature = 23 ± 2 °C. Error bars represent standard deviation between parallel experiments. Dash lines represent kinetic modeling for POAs by a Hom model with *k*_obs_ in Table S4.

Disinfection experiments showed that the inactivation efficiency for *Escherichia coli* and *Staphylococcus epidermidis* followed the order of PFA > chlorine > PAA ≈ PPA (oxidant dosage = 120 μM (within the range of practical application in wastewater treatment plants)^57^), at pH 7.1 or 7.8 (two environmentally relevant pHs with different oxidant speciation) (Figure 1). The inactivation kinetics of POAs were well-fitted by the Hom model (Table S4) as suggested in previous research (eq 5).^29^
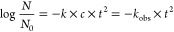
5where *N* is the cell density and *N*_0_ is the initial cell density (in CFU·mL^–1^); *c* is the oxidant concentration, which is assumed to remain at the initial concentration (120 μM); *t* is the reaction time (in min); *k* and *k*_obs_ are the rate constant and observed rate constant (in M^–1^·min^–2^ and min^–2^), respectively. As discussed, PAA and PPA decayed negligibly in the AnMBR matrix. Although PFA self-decay was observed, its self-decay was less than 8% within the reaction time for kinetic modeling (1 min). Thus, we can assume *c* = *c*_0_ for all POAs in the kinetic modeling. The fitting of POA inactivation kinetics into the Hom model (proportional to *t*^2^) suggests that the loss of culturability may require accumulated damage by POAs.

For *E. coli* inactivation, PFA exhibited a *k*_obs_ at 2.93–1.03 min^–2^, while PAA and PPA only resulted in *k*_obs_ at 0.28–0.30 min^–2^, at pH 7.1 and 7.8 where the pH impact was minor (Table S3). The inactivation of *S. epidermidis* was faster than that of *E. coli*, where PFA had *k*_obs_ at 4.38 (pH 7.1) and 3.42 (pH 7.8) min^–2^, and PAA/PPA yielded *k*_obs_ at 0.60–0.63 min^–2^. The increase of pH from 7.1 to 7.8 considerably inhibited the disinfection of *S. epidermidis* by PFA but did not affect the performance of PAA and PPA (Figure 1). With a p*K*a at 7.3, the percentage of protonated PFA decreases sharply from 61.3% at pH 7.1 to 24.0% at pH 7.8.^55^ However, the pH effect was moderate for PAA and PPA due to their higher p*K*a values. For PAA (p*K*a 8.2),^46^ the protonated percentage is 92.6% and 71.5% at pH 7.1 and 7.8, respectively. The p*K*a for PPA is not available from the literature but expected to be similar to or higher than that of PAA. Despite this pH effect, PFA outperformed PAA and PPA at both pHs in the inactivation of the two bacteria, while the performance of PAA and PPA was similar.

Spiking of NaOCl (at the same concentration as POA) resulted in more complex disinfection kinetics that could not fit the Hom model (Figure 1), which is probably due to the complicated self-decay and copresence of free and combined chlorine. Overall, chlorine achieved 3.2 ± 0.2 and 3.4 ± 0.6 log removal of *E. coli* in 2 min at pH 7.1 and 7.8, respectively, which was between the performance of PFA and PAA. The 2 min of inactivation of *S. epidermidis* by chlorine was between PAA/PAA and PFA, probably due to chlorine consumption by the water matrix. Generally, free chlorine is a stronger and less selective oxidant than POAs and, hence, is expected to inactivate bacteria faster. However, wastewater effluents (especially AnMBR effluents) contain a considerable level of ammonia,^17^ which converts free chlorine to the less reactive monochloramine.^3^ Furthermore, EfOM rich in amine and phenolic moieties may consume available chlorine rapidly, leading to a slower bacteria inactivation.^8,9^ Nonetheless, the bacterial inactivation efficiency of PAA in AnMBR effluent (this study) is similar to that in phosphate buffer in our previous study, indicating that the wastewater matrix may have only a mild effect on POA disinfection.^29^ Therefore, dosing of free chlorine often exhibits similar or less effective wastewater disinfection performance than POAs.^8,9^

Generally, the results are consistent with previous research in that (1) PFA achieved more efficient inactivation than PAA^10,11,15^ and (2) chlorine performed similarly or less efficiently than PFA but better than PAA in bacteria inactivation.^8,9^ Furthermore, as carboxylic acids are used for medical disinfection,^58,59^ the effects of formic acid, acetic acid, and propionic acid on bacteria culturing were investigated using phosphate buffer at pH 7.1. None of the carboxylic acids led to significant loss (>1 order of magnitude) of culturability of the bacteria (Figure S3). In addition, our previous studies evaluated and confirmed that the coexistent H_2_O_2_ in PAA solution had a negligible contribution to inactivating *E. coli* and *S. epidermidis*.^28,29^ Hence, the disinfection was mainly attributed to oxidation by POAs, with minimum impact of coexisting carboxylic acids and H_2_O_2_.

### 3.2 Bacterial Inactivation Mechanisms: Fluorescence Microscopy Study

**Figure 2:**
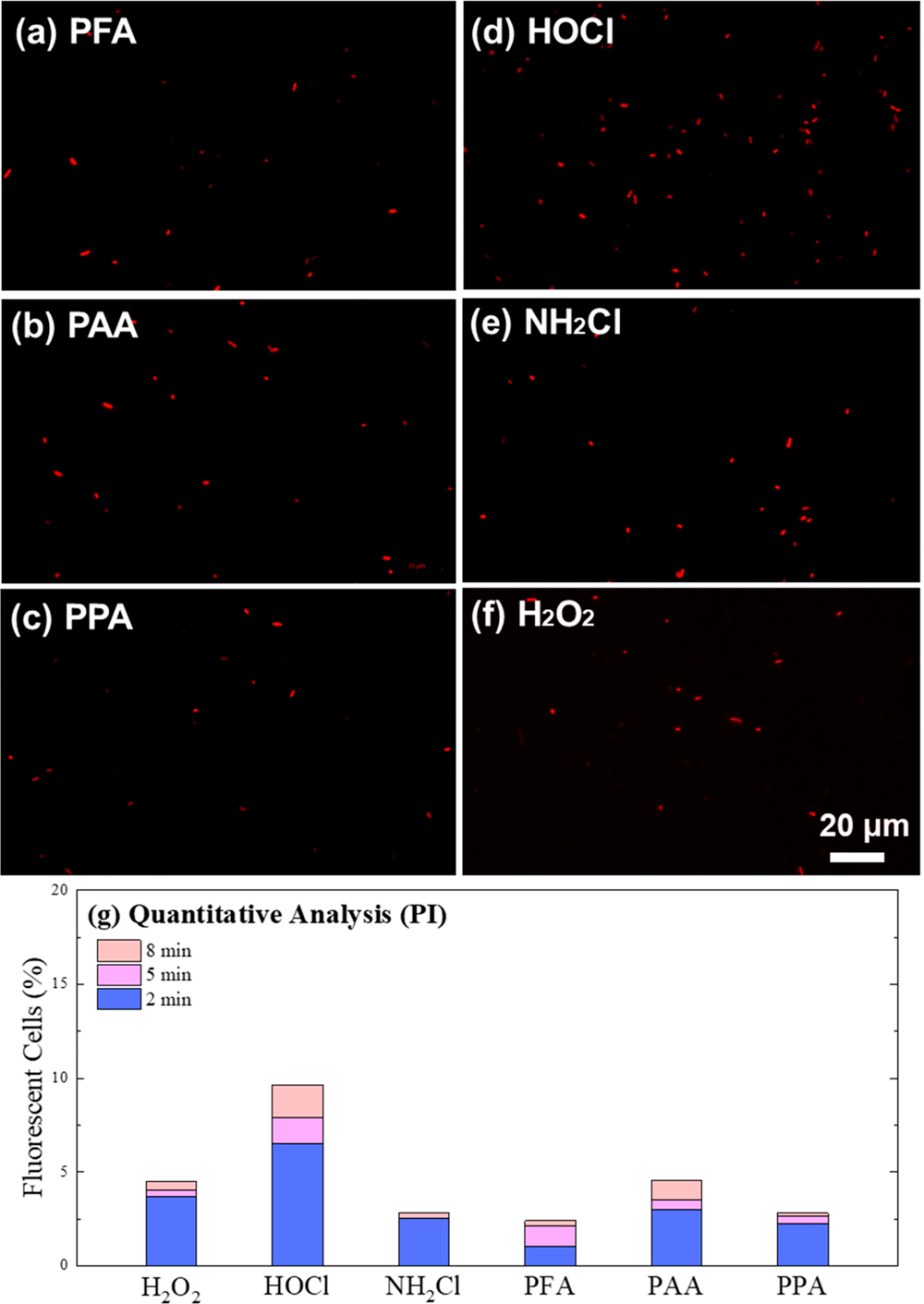
(a–f) Fluorescence microscope images of PI-incubated *E. coli* treated by different oxidants for 8 min and (g) the quantitative analysis. Experimental conditions: [oxidants]_0_ = 120 μM, [PI]_0_ = 15 μM, pH = 7.1, [phosphate buffer] = 10 mM, temperature = 23 ± 2 °C.

To further understand the bacterial inactivation mechanisms of the oxidants, DCFH-DA- and PI-colored *E. coli* were observed under a fluorescence microscope, and the cell counts were estimated by MATLAB. PI can only permeate cells that have lost membrane integrity and develop a red color fluorescence.^21,22^ As shown in Figure 2 and Figures S4–S9, minimal increase in red fluorescent cell numbers (<5% of total cells) was observed during the 8 min oxidation by POAs, H_2_O_2_, and monochloramine, indicating their low capacity in damaging cell membranes. In contrast, obvious red fluorescence (∼6.52%) was developed as soon as 2 min after free chlorine oxidation (Figure S8), indicating rapid membrane damage and cell lysis.

**Figure 3:**
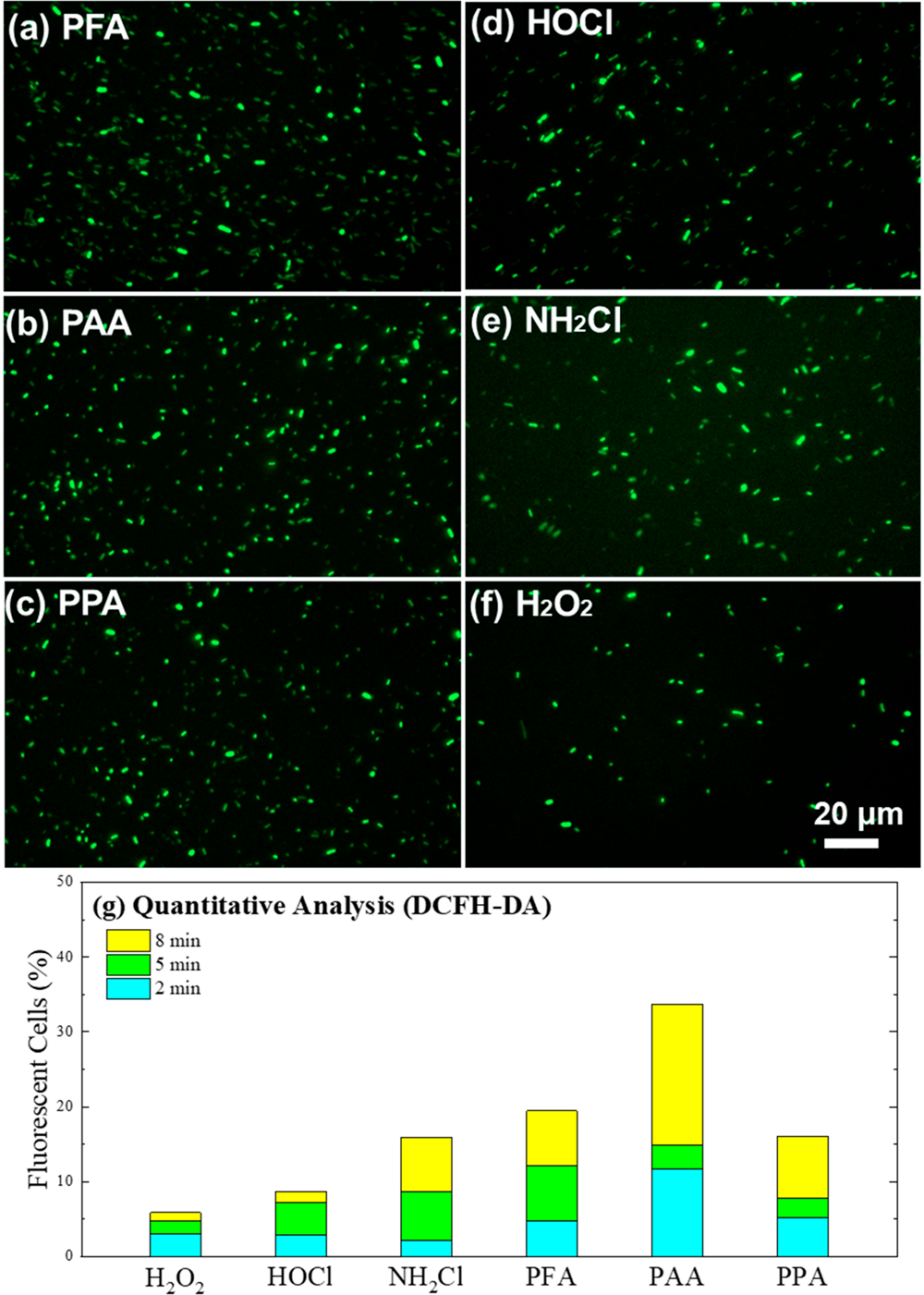
(a–f) Fluorescence microscope images of DCFH-DA-incubated *E. coli* treated by different oxidants for 8 min and (g) the quantitative analysis. Experimental conditions: [oxidants]_0_ = 120 μM, [DCFH-DA]_0_ = 340 μM, preincubation time = 30 min, pH = 7.1, [phosphate buffer] = 10 mM, temperature = 23 ± 2 °C.

DCFH-DA is a widely used fluorescent indicator for intracellular oxidative stress.^29^ Briefly, DCFH-DA can be hydrolyzed inside the cells to produce DCFH, which generates fluorescent 2,7-dichlorofluorescein (DCF) with reactive oxygen species (ROS) or oxidants, indicating oxidant accumulation inside the cells. In the DCFH-DA experiments, the green fluorescent intensity (representing intracellular oxidative stress) during oxidation follows the order of PAA > PFA ≈ PPA ≈ monochloramine > free chorine > H_2_O_2_ (Figure 3).

Despite of its rapid damage of bacteria membranes, free chlorine led to slightly weaker intracellular oxidative stress. Notably, unlike POAs (Figures S4–S6), free chlorine caused negligible fluorescence (∼2.87% of total cells) in the DCFH-DA experiment at 2 min, while significant cell lysis (indicated by the PI experiment) was already achieved within the same time period (Figure 3g and Figure S8). In other words, free chlorine induced rapid cell lysis, rather than intracellular oxidation, which could be explained by the consumption by EPS and cell components due to the less selective oxidation of free chlorine.^22,27,60^ In contrast, POAs likely penetrate intact cell membranes and accumulate intracellularly without lysing the cells.

Finally, H_2_O_2_, at the same concentration, also led to very limited intracellular oxidative stress (∼5.85% fluorescent after 8 min), probably due to the enzymatic decomposition of H_2_O_2_. It is well-known that bacteria cells synthesize ROS-scavenging enzymes, such as superoxide dismutase (SOD) and peroxidase (POD), to enable the chain reactions for ROS and H_2_O_2_ scavenging.^61^ Considering that cell membrane is theoretically permeable to H_2_O_2_ due to its smaller molecular weight than POAs, it is most plausible that H_2_O_2_ diffused into the cells but was consumed by ROS-scavenging enzymes and, hence, could not be accumulated. Unlike H_2_O_2_, POAs appear to be resistant to decomposition by intracellular ROS-scavenging enzymes, which may be due to POAs’ selective reactivity to oxidize some amino acids (e.g., cysteine) of the enzymes (see more discussion later).^62^

To sum up, (1) POAs penetrate intact cell membranes and resist oxidant-scavenging enzymes and, hence, are accumulated inside the cells; (2) free chlorine induces rapid cell surface damage and lysis; meanwhile, chlorine is consumed by EPS and cell components before intracellular accumulation; and (3) H_2_O_2_ likely permeates through cell membranes but is decomposed by ROS-scavenging enzymes prior to accumulation. Therefore, POAs can lead to a higher intracellular oxidant level than the other oxidants without severe cell lysis.

The unique interaction of POAs with bacteria could be a double-edged sword. On one hand, POA can inactivate bacteria without releasing IPS and iARG. On the other hand, POA disinfection may leave the bacteria in a VBNC status, which may allow for microbial regrowth. A regrowth test was thus conducted, and no significant regrowth of POA-inactivated bacteria was found after 48 h of incubation (Figure S10), confirming a low risk of regrowth.

### 3.3 Inactivation of Viruses in Phosphate Buffer

Viral inactivation by POAs was studied in clean phosphate buffer due to the difficulties of virus culturing in bacteria-rich effluents and the knowledge gaps on POA-inactivation of viruses in clean matrix. First, the self-decay of oxidants in clean phosphate buffer was studied. It was found that the self-decay of PAA and free chlorine was negligible over 30 min in pH 7.1 phosphate buffer (data not shown). In contrast, the self-decay of PFA was significant at all three tested pH values (5.5, 7.1, and 7.8) and not significantly affected by different initial PFA concentrations (50–200 μM) (Figure S11) or the presence of viruses (data not shown). The self-decay of PFA was a first-order reaction when [PFA] < 200 μM (eqs 3 and 4).^55^

Then, the cumulative exposure to PFA (C·T value) could be calculated by a pseudo-first-order model (eqs 6 and 7), while the C·T values for PAA and free chlorine were simply calculated by multiplying their initial concentration and the reaction time.
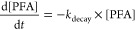
6
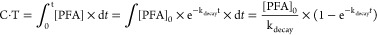
7*k*_decay_, the first-order rate constant for PFA self-decay, was calculated to be 0.033 ± 0.003, 0.047 ± 0.003, and 0.074 ± 0.003 min^–1^ at pH 5.5, 7.1, and 7.8, respectively.

**Figure 4:**
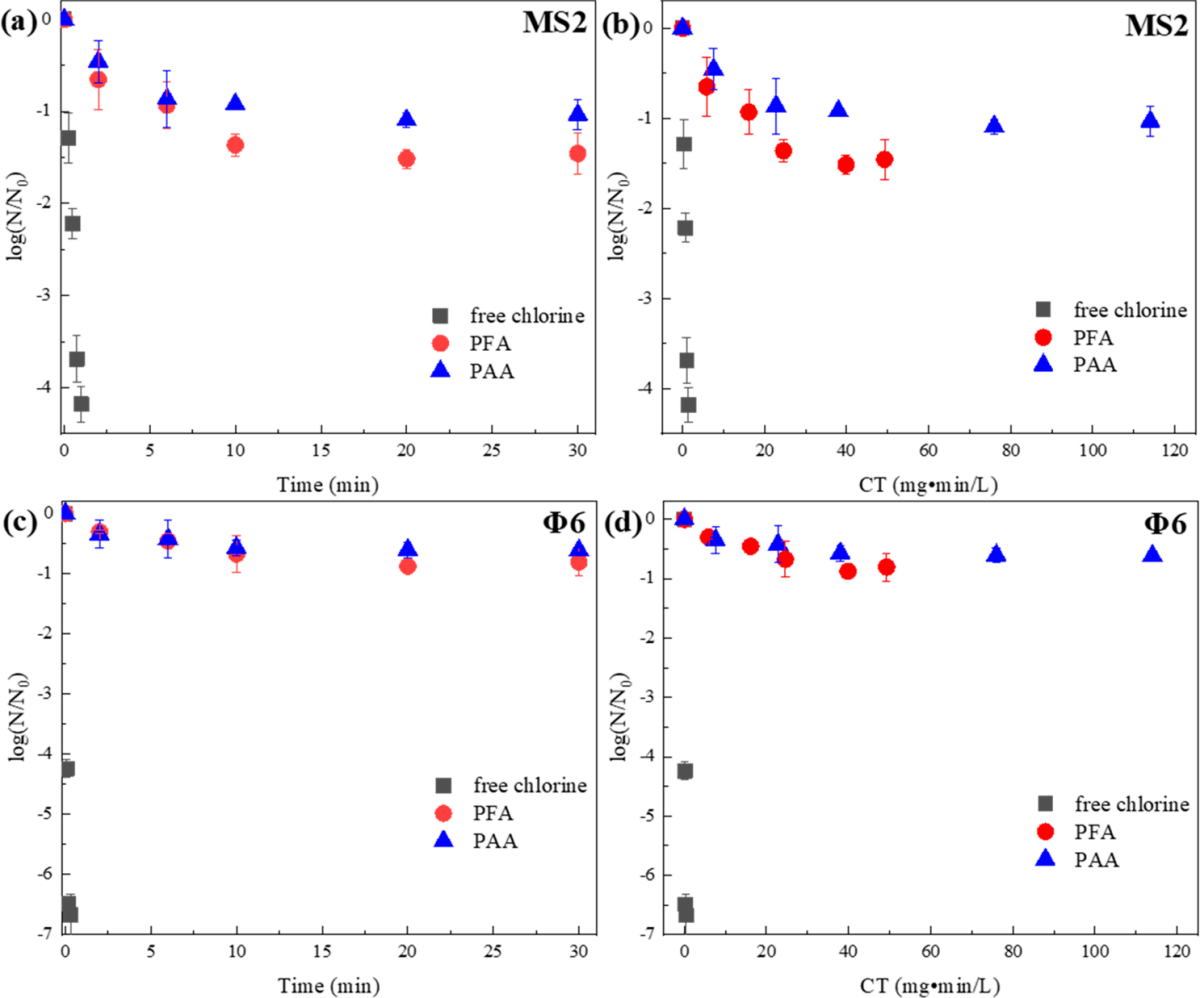
Inactivation of MS2 and Φ6 in phosphate buffer. Experimental conditions: [PFA]_0_ = 50 μM (3.1 mg·L^–1^), [PAA]_0_ = 50 μM (3.8 mg·L^–1^), [free chlorine]_0_ = 20 μM (1.42 mg Cl_2_·L^–1^), [MS2]_0_ = 1 × 10^6^ PFU·mL^–1^, [Φ6]_0_ = 1 × 10^8^ PFU·mL^–1^, pH = 7.1, [phosphate buffer] = 10 mM, temperature = 23 ± 2 °C. Error bars represent standard deviation between triplicate experiments.

**Figure 5:**
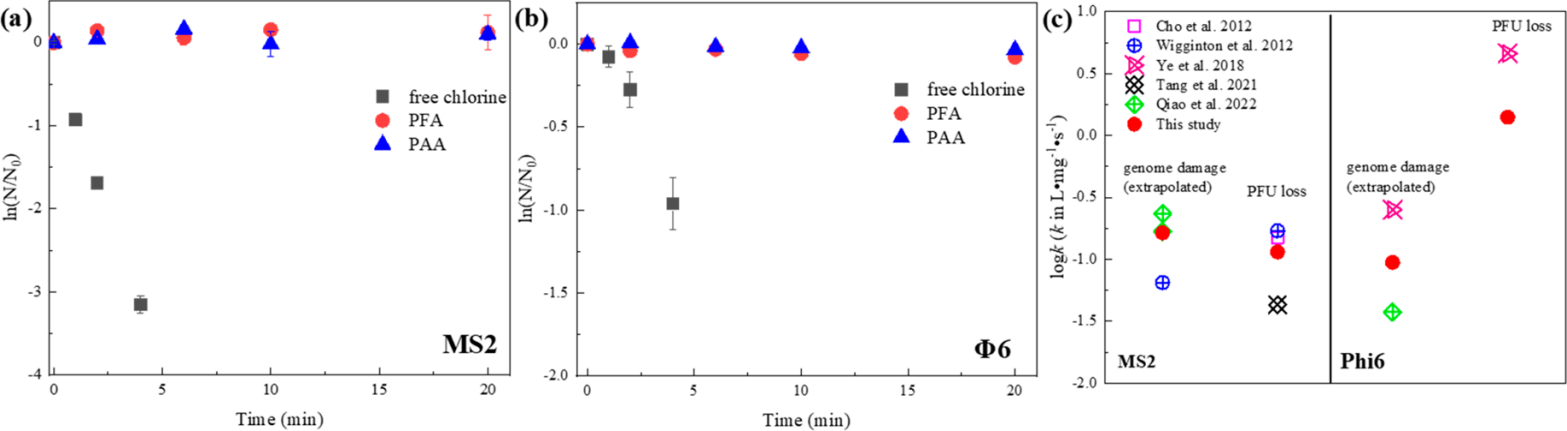
Degradation of the genomes of MS2 (a, target sequence = 83 bases) and Φ6 (b, target sequence = 140 base pairs = 280 bases) with oxidants in DI water with phosphate buffer, and (c) predicted entire genome damage rates versus observed PFU loss rates by free chlorine. Experimental conditions for genome damage experiments: [PFA]_0_ = 100 μM (6.2 mg·L^–1^), [PAA]_0_ = 100 μM (7.6 mg·L^–1^), [free chlorine]_0_ = 50 μM (3.55 mg Cl_2_·L^–1^), [MS2]_0_ = 1 × 10^6^ PFU·mL^–1^, [Φ6]_0_ = 1 × 10^8^ PFU·mL^–1^, pH = 7.1, [phosphate buffer] = 10 mM, temperature = 23 ± 2 °C. Error bars represent standard deviation between duplicate measurements in the RT-qPCR.

MS2 (nonenveloped virus) inactivation by PFA, PAA, and free chlorine was compared at pH 7.1 (Figure 4a,b). Consistent with the earlier study by Dunkin et al.,^31^ MS2 showed great resistance to PAA oxidation, with only ∼1-log PFU removal after 30 min of disinfection (114 mg·min·L^–1^ C·T exposure). For the first time, this study revealed that MS2 inactivation by PFA was as inefficient as that of PAA, with ∼1.4-log PFU removal after 30 min of exposure (Figure 4a,b). Furthermore, the disinfection by PFA and PAA both reached an early plateau, indicating that most inactivation was achieved within the first 6 min and extended exposure could not result in additional inactivation. In contrast, free chlorine led to a rapid inactivation with a pseudo-first-order rate constant (*k*_PFU_, with regard to C·T exposure) at 0.13 L·mg^–1^·s^–1^, which is consistent with several previous studies (Figure 5c).^39,63,64^ These results are in sharp contrast with the bacterial inactivation experiments, where POAs showed comparable inactivation capacity as chlorine.

Φ6, representing the enveloped viruses, was evaluated to compare the disinfection performance of PFA, PAA, and free chlorine (Figure 4c,d). Until this work, POA inactivation of enveloped viruses has been rarely studied. Generally, enveloped viruses are more susceptible to oxidation.^37^ Surprisingly, we found Φ6 to exhibit greater resistance to POAs than MS2, where both PFA and PAA resulted in less than 1-log PFU removal after 30 min of reaction. In contrast, free chlorine inactivated Φ6 to near the detection limit (i.e., < 5 PFU on the plates without any dilution of the samples, ∼7-log PFU removal in our experimental conditions) in less than 20 s (Figure 4c). Φ6 inactivation by free chlorine is remarkably faster than that of MS2 with a *k*_PFU_ at 1.51 L·mg^–1^·s^–1^. These kinetics were slower than but on the same order of magnitude with that reported by Ye et al. (4.6 L·mg^–1^·s^–1^) (Figure 5c), possibly because of the different reactor setup or initial chlorine concentrations.^36^

In summary, this study revealed the high resistance of MS2 and Φ6 to POA oxidation, regardless of the viral structure (enveloped or nonenveloped) and POA type (PFA or PAA). Interestingly, unlike typical oxidants (e.g., chlorine and ozone) that exhibit the order of inactivation efficiency as enveloped viruses > nonenveloped viruses > bacteria,^42,65,66^ POAs are most effective toward bacteria while facing strong resistance from both enveloped and nonenveloped viruses. It should be noted that research has shown that PAA could oxidize halides to free halogen (e.g., HOCl, HOBr) for viral inactivation; hence, POAs may exhibit stronger inactivation for viruses in some real water matrixes.^67^

### 3.4 Genome Damage of the Viruses

The damage of the viral genomes was studied by RT-qPCR. Interestingly, we found PFA and PAA led to negligible loss of target sections of the genomes for both bacteriophages (Figure 5a,b). Thus, the inactivation of the two viruses by POAs, albeit modest, should be attributed to loss of protein functionalities. Moreover, we anticipate the POA-inactivated viruses to remain detectable by RT-qPCR methods, which has already been reported for surface disinfection of SARS-CoV-2 by PAA.^68^ Contrary to POAs, free chlorine led to a significant genome loss with normalized rate constants (*k*_normalized_, eqs 1 and 2) at 4.55 and 0.35 L·mg^–1^·s^–1^·base^–1^, for MS2 and Φ6, respectively, which are comparable with previous publications (Figure 5c).^36,39,69,70^ The *k*_normalized_ for the enveloped Φ6 was significantly lower than that of the nonenveloped MS2, which could be ascribed to the stability of dsRNA relative to ssRNA, or the shielding effect of the glycerophospholipid envelope.^69^

The genome damage by free chlorine was extrapolated to the entire genome by eqs 1 and 2, with the assumption that the reactivity of the targeted and untargeted genome sections is similar. The entire genome damage rate (*k*_genome_, eqs 1 and 2) of MS2 by free chlorine was 0.15 L·mg^–1^·s^–1^, similar to its infectivity loss rate (*k*_PFU_), suggesting that MS2 inactivation by free chlorine was driven by genome destruction, concurring with Wigginton et al.^39^ In contrast, the genome loss only accounted for 6.62% (*k*_genome_*/k*_PFU_) of the Φ6 inactivation by free chlorine, suggesting that Φ6 inactivation was dominated by protein damage, which is consistent with Ye et al.^36^

### 3.5 Oxidation of Amino Acids and Ribonucleotides by POAs

To further understand the POA inactivation mechanisms, the reactivity of POAs with amino acids and ribonucleotides was assessed. Previous studies have reported that PAA only reacts with cysteine and methionine, among all the tested amino acids and ribonucleotides.^45,62^ Therefore, we studied the reactions of PAA with cysteine and methionine using a quenched flow system that was described previously.^47^ However, 100 μM PAA was totally consumed by equimolar cysteine or methionine within 0.15 s, suggesting that the rate constants between PAA and these two amino acids are greater than 1 × 10^5^ M^–1^·s^–1^, the upper limit of rate constants that could be determined by the quenched flow system setup.^47^

We also investigated the reactions of PFA and PPA with biomolecules by monitoring POA decay with the amino acids/ribonucleotides in excess and applying the pseudo-first-order model to calculate the rate constants (eqs 8 and 9).
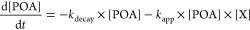
8
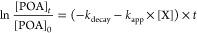
9where [X] is the initial concentration of the biomolecule (in M), *k*_decay_ is the self-decay rate constant of the POA (in s^–1^), *k*_app_ is the second-order rate constant between PFA or PPA with the biomolecule (in M^–1^·s^–1^), and *t* is the reaction time (in s). As shown in Table 1, 10 of the 12 amino acids exhibited *k*_app_ values lower than 0.3 M^–1^·s^–1^ with PFA or PPA (mostly <0.1 M^–1^·s^–1^). The oxidation products of selected amino acids were studied by liquid chromatography–high-resolution mass spectrometry (LC–HRMS), with details described by Du et al. (Text S7).^62^ Similar to PAA, PFA and PPA only exhibited high reactivity with cysteine and methionine, through an oxygen-transfer pathway as identified previously (Figure S12).^62^ The reactivity of PFA and PPA with four ribonucleotides was also very low (Table 1), consistent with the little genome damage by PFA oxidation. In contrast, free chlorine has high reactivity toward most of the biomolecules, inducing less selective damage on the genomes and proteins of the viruses.

**Table 1:** Second-Order Rate Constants between Biomolecules and Disinfectants^a^

			apparent second-order rate constant (M^–1^·s^–1^)			
No.	compounds	PFA (pH = 5.5)	PFA (pH = 7.1)	PAA (pH = 7.0)	PPA (pH = 7.0)	free chlorine (pH = 7.0)
amino acids						
1	glycine	<0.1	<0.1	slow^45^	<0.1	1.5 × 10^5^^3^
2	proline	<0.1	<0.1	slow^45^	<0.1	3.5 × 10^3^^3^
3	glutamic acid	<0.1	<0.1	slow^45^	<0.1	
4	aspartic acid	<0.1	<0.1	slow^45^	<0.1	
5	tyrosine	0.130	<0.1	slow^45^	<0.1	4.4 × 10^1^^74^
6	tryptophan	0.225	<0.1	slow^45^	<0.1	1.1 × 10^4^^74^
7	serine	<0.1	<0.1	slow^45^	<0.1	1.7 × 10^5^^3^
8	cysteine	>1 × 10^5^	>1 × 10^5^	>1 × 10^5^	>1 × 10^5^	3.0 × 10^7^^74^
9	methionine	>1 × 10^5^	>1 × 10^5^	>1 × 10^5^	>1 × 10^5^	3.8 × 10^7^^74^
10	lysine	<0.1	<0.1	slow^45^	<0.1	5.0 × 10^3^^74^
11	arginine	<0.1	<0.1	slow^45^	<0.1	2.6 × 10^1^^3^
12	histidine	0.294	<0.1	1.8^62^	0.833	1.0 × 10^5^^74^
ribonucleotides						
1	adenosine monophosphate	<0.1	<0.1	slow^45^	<0.1	6.4^3^
2	guanosine monophosphate	<0.1	<0.1	slow^45^	<0.1	2.1 × 10^4^^3^
3	uridine monophosphate	<0.1	<0.1	slow^45^	<0.1	5.5 × 10^3^^3^
4	cytidine monophosphate	<0.1	0.254	slow^45^	<0.1	6.6 × 10^1^^3^

a Note: The rate constants without notation for references were acquired in this study.

The above results are consistent with the outcomes of bacterial and viral inactivation experiments in this study. Briefly, POAs primarily inactivate pathogens through oxidation of cysteine and methionine, while their reactivity with other biomolecules is limited. Unlike free chlorine, which oxidizes with low selectivity, is consumed by cell surfaces, and leads to membrane damage/cell lysis, the three POAs are intracellularly accumulated without severe cell surface damage.

**Figure 6:**
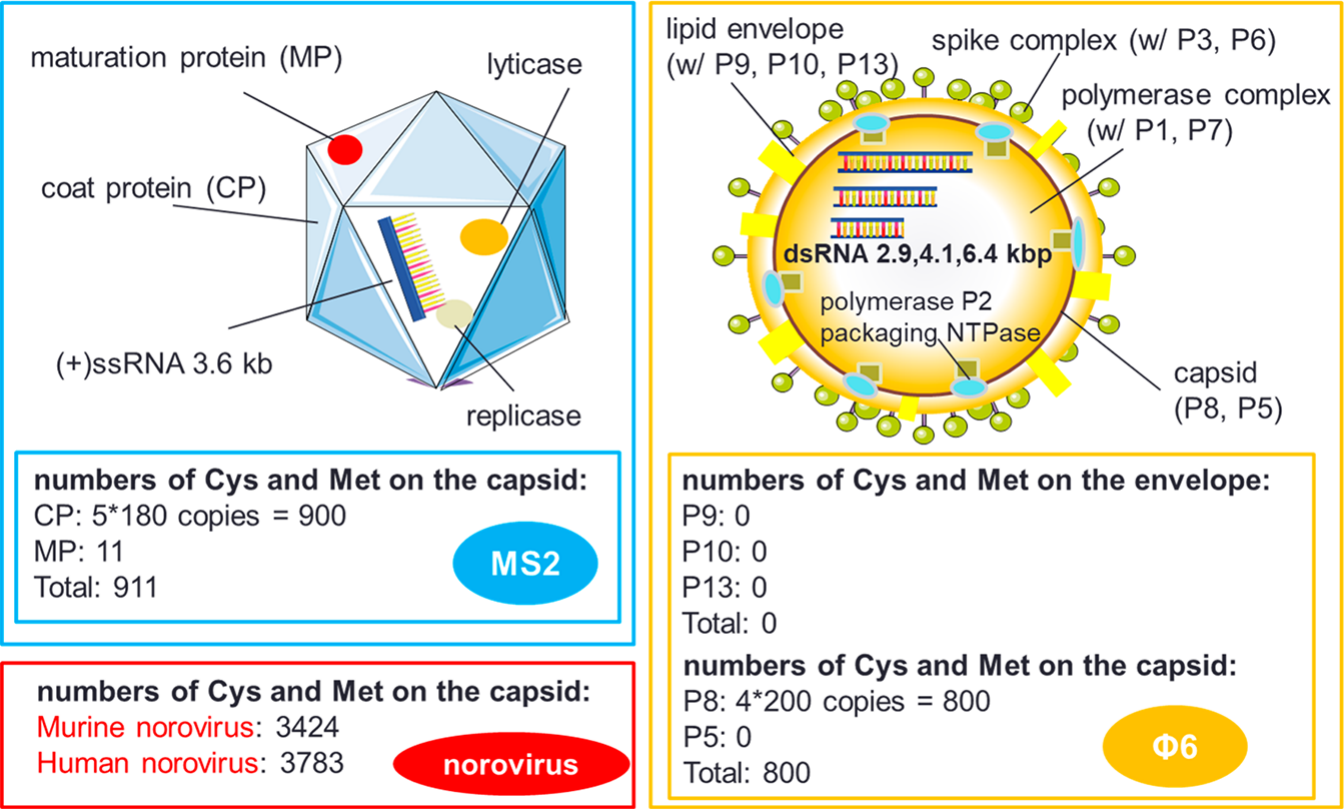
Structure comparison of MS2 and Φ6. Cys and Met are abbreviations for cysteine and methionine, respectively. The data for MS2 was retrieved from Schmitz et al.^45^ after confirmation, and the data for Φ6 was retrieved from (www.uniprot.org).

As for the viruses, their structures have been well-studied and their protein sequences on the surface of the viruses were retrieved from the Universal Protein Resource database (www.uniprot.org). MS2 is a nonenveloped icosahedral bacteriophage that consists of four proteins and the ssRNA genome (Figure 6).^39^ Its capsid is composed of 180 copies of coat proteins and 1 copy of maturation protein.^45^ Φ6 is composed of one envelope, two concentric protein layers, and three segments of linear dsRNA encoding 13 proteins (P1–P13) (Figure 6).^71^ The phospholipid envelope contains proteins P9, P10, and P13, with the host attachment spike proteins (P3) anchored via fusogenic protein (P6). Its capsid contains 200 copies of protein P8 trimers and the lytic enzyme P5. As demonstrated by Schmitz et al.,^45^ each cysteine or methionine on the surface was counted as one POA-reactive site. To sum up, MS2 and Φ6 both lack POA-reactive sites on their surfaces; hence, they are difficult to be inactivated by POAs, regardless of the envelope structure. PFA could not inactivate PAA-resistant viruses more efficiently due to its similar chemical selectivity as PAA, even though PFA showed better performance for the PAA-reactive pathogens.

## 4 Environmental Implications

This study provides several first-time data and information that will be very useful for future research on POAs. Reproducible lab-scale synthesis methods for PFA and PPA were developed (Text S2), and POAs and chlorine were systematically evaluated for bacterial and viral inactivation. POAs exhibit similar disinfection efficiency as chlorine for bacteria; however, their viral inactivation capacity is limited, regardless of the envelope structure.

This study demonstrates distinctively different inactivation mechanisms of POAs versus chlorine, which is attributed to their different reactivity to biomolecules. POAs selectively react with cysteine and methionine, while free chlorine is reactive toward most amino acids and nucleotides. For bacteria, POAs result in more intracellular accumulation than free chlorine without severe damage of cell surfaces, and the coexistent H_2_O_2_ had a minimal contribution for the intracellular oxidant levels. Therefore, POAs may have promising potential in controlling cell lysis and IPS/ARG release. For example, free chlorine can trigger IPS release, which in turn leads to aggravated membrane fouling and DBP formation, during membrane cleaning of MBRs.^20,60,72^ Thus, POAs that inactivate bacteria intracellularly should be considered for in situ membrane cleaning and biofouling control in MBRs. Moreover, UV/POAs may outperform UV/H_2_O_2_ and UV/chlorine for pathogen inactivation, due to the photogeneration of radicals inside the cells, which has been demonstrated with PAA.^28,29,73^

For viruses, this study revealed that the protein sequence, rather than the envelope structure, is important for PFA/PAA disinfection efficiency. Thus, cysteine and methionine content could be more important than structure similarity for surrogate selection in POA disinfection studies. Even though PFA achieved faster inactivation of the bacteria, it could hardly inactivate the PAA-resistant viruses (MS2 and Φ6). The unsatisfactory viral inactivation (particularly those lacking cysteine and methionine) could be a problem for POA disinfectants. Therefore, research on combined disinfection (e.g., UV/PAA^29^) should be conducted to enhance the viral inactivation in POA-based disinfection.

Overall, this study identifies the unique pathogen inactivation performance of POAs versus conventional chlorine and provides highly valuable insights for broadening the suitability of POAs in (waste)water disinfection and other microbial control applications.

## Supporting Information Available

Discussions of chemicals, reagents, and microbial cultures, synthesis of PFA and PPA, wastewater sample, bacteria and virus measurements, genome damage measurement, binding assay, and chemical analysis methods, tables of comparison of three POAs, AnMBR synthetic wastewater feed composition, wastewater effluent characterization, and observed disinfection rate constants of three POAs, and figures of ABTS^•+^ production from PAA and H_2_O_2_ in the presence of HRP, decay of disinfectants in the effluent from a bench-scale AnMBR, effects of formic acid, acetic acid, and propionic acid on bacteria growth, fluorescence microscope images, bacteria regrowth tests, PFA decay, structures of selected amino acids, ribonucleotides, oxidation products of cysteine, and methionine, and LC–HRMS peaks for the oxidation products of cysteine and methionine (PDF)
