# Supplementary material for: Bacteria and Virus Inactivation: Relative Efficacy and Mechanisms of Peroxyacids and Chlor(am)ine
Source: Environ Sci Technol. 2023 Mar 30;57(47):18710–21. doi: 10.1021/acs.est.2c09824 (PMC10690719; doi:10.1021/acs.est.2c09824)
Supplement: Supplementary file 1 — es2c09824_si_001.pdf [file es2c09824_si_001.pdf]

Supporting Information

Bacteria and Virus Inactivation: Relative Efficacy and Mechanisms of Peroxyacids and Chlor(am)ine

Junyue Wang,[†] Wensi Chen,[†] Ting Wang,[†] Elliot Reid,[†] Caroline Krall,[†] Juhee Kim,[†] Tianqi Zhang[‡], Xing Xie,[†] Ching-Hua Huang*[,†]

[†]School of Civil and Environmental Engineering, Georgia Institute of Technology, Atlanta, Georgia 30332, United States

[‡]School of Architecture, Civil and Environmental Engineering (ENAC), École Polytechnique Fédérale de Lausanne (EPFL), 1015, Lausanne, Switzerland

*Corresponding Author.

E-mails: ching-hua.huang@ce.gatech.edu (Ching-Hua Huang)

Number of Pages: 28

Number of Texts: 7

Number of Tables: 4

Number of Figures: 13

Number of References: 15

Text S1. Chemicals, Reagents, and Microbial Cultures

Sodium hydroxide (NaOH), sulfuric acid (H_2SO_4), sodium thiosulfate ($Na_2S_2O_3$), potassium iodide (KI), *N,N*-diethyl-*p*-phenylenediamine (DPD), 2,2'-azino-bis(3-ethylbenzothiazoline-6-sulfonic)acid (ABTS), formic acid, acetic acid, propionic acid, and Na_2HPO_4 (>99%) were purchased from Sigma-Aldrich or Fisher Scientific. Twelve amino acids and four ribonucleotides were used to elucidate the second-order rate constants and reaction mechanisms of PFA with biomolecules, all purchased at the highest purity from Sigma-Aldrich (structures see Figure S12). Deionized water (DI water) (>18 mΩ-cm) was produced from a Milli-Q water purification system (Billerica, MA). The phosphate buffer (100 mM) stock solution was prepared by dissolving Na_2HPO_4 (>99%) in to water and adjust the pH by NaOH and H_2SO_4, and diluted to 10 mM in batch disinfection experiments.

Escherichia coli (ATCC 10798) and *Staphylococcus epidermidis* (*S. epidermidis*) (ATCC 12228) were purchased from ATCC, and revived in DifcoTM LB and Difco nutrient broth (VWR International Inc. (Radnor, PA)), respectively, for ~18-h growth at 37 °C.[1] One mL of the as-prepared culture was centrifuged at 4000 rpm for 5 min. Then, the pellet was washed by DI water for three times and finally re-suspended in 1-mL DI water.

Bacteriophage MS2 (ATCC 15597-B1) and its host bacterium *Escherichia coli* (*E. coli*, ATCC 15597) were purchased from ATCC. Φ6 as well as its host bacterium *Pseudomonas syringae* (*P. syringae*) were kindly provided by Dr. Yinyin Ye's lab at the University of New York at Buffalo. MS2 and Φ6 were inoculated into 20 mL of tryptic soy broth (TSB, 30 $g \cdot L^{-1}$), containing ~10^7 $CFU \cdot mL^{-1}$ each of *E. coli* and *P. syringae*, respectively. The as-prepared MS2 and Φ6

solutions were sealed in centrifuge tubes and incubated overnight (~16 h) at 35 °C and 25 °C, respectively. Before the experiments for both viruses, the suspension was centrifuged at 4000 rpm for 5 min, and the supernatant was filtered through a 0.2 μm syringe filter to remove the host bacteria.

Text S2. Synthesis of PFA and PPA

PFA synthesis. PFA was synthesized by mixing 1.5 mL of H_2O_2 (30% w/w), 2.5 mL of formic acid (96% purity), and 0.2 mL of H_2SO_4 at 40 °C for 8 min. The density, purity, and molecular weight of formic acid was reported by the manufacture. The concentration of the H_2O_2 stock was measured by titration methods as described in our previous study.[2] The generated PFA was stored at -4 °C and used within 2 h. The concentrations of formed PFA and coexistent H_2O_2 in the PFA solution were measured as described in Text S7. The conversion rates of formic acid and H_2O_2 to yield PFA were calculated as following:

$$[\text{formic acid}]_0 = 0.96 \times \frac{1.22\ \text{g/ml}}{46\ \text{g/mol}} \times \frac{2.5\ \text{mL}}{4.2\ \text{mL}} = 15.15\ \text{M} \quad \text{(S1)}$$

$$[H_2O_2]_{\text{stock}} = 8.85\ \text{M (measured)}, [H_2O_2]_0 = 8.85\ \text{M} \times \frac{1.5\ \text{mL}}{4.2\ \text{mL}} = 3.16\ \text{M} \quad \text{(S2)}$$

$$[\text{PFA}]_{\text{final}} = 1.976\ \text{M (measured)}, [H_2O_2]_{\text{final}} = 1.126\ \text{M (measured)} \quad \text{(S3)}$$

$$\text{conversion (formic acid)} = \frac{1.976\ \text{M}}{15.15\ \text{M}} = 13.04\% \quad \text{(S4)}$$

$$\text{conversion } (H_2O_2) = \frac{1.976\ \text{M}}{3.16\ \text{M}} = 62.53\% \quad \text{(S5)}$$

PPA synthesis. PPA was synthesized by mixing 1.0 mL of H_2O_2 (30% w/w), 2.0 mL of propionic acid (98% purity), and 0.2 mL of H_2SO_4 at 40 °C for 15 min. The density, purity, and molecular weight of propionic acid was reported by the manufacture. The concentration of the H_2O_2 stock was measured as described above. The generated PPA was stored at -4 °C for no more than 1 week, during this period the PPA concentration was not changed (data not shown). The concentrations of formed PPA and coexistent H_2O_2 in the PPA solution were measured as described in Text S7. The conversion rates of propionic acid and H_2O_2 to yield PPA were calculated as following:

$$[\text{propionic acid}]_0 = 0.98 \times \frac{0.99\ \text{g/mL}}{74\ \text{g/mol}} \times \frac{2\ \text{mL}}{3.2\ \text{mL}} = 8.194\ \text{M} \tag{S6}$$

$$[\text{H}_2\text{O}_2]_{\text{stock}} = 8.85\ \text{M (measured)}, [\text{H}_2\text{O}_2]_0 = 8.85\ \text{M} \times \frac{1\ \text{mL}}{3.2\ \text{mL}} = 2.76\text{M} \tag{S7}$$

$$[\text{PPA}]_{\text{final}} = 1.689\ \text{M (measured)}, [\text{H}_2\text{O}_2]_{\text{final}} = 1.064\ \text{M (measured)} \tag{S8}$$

$$\text{conversion (propionic acid)} = \frac{1.689\ \text{M}}{8.194\ \text{M}} = 20.61\% \tag{S9}$$

$$\text{conversion } (\text{H}_2\text{O}_2) = \frac{1.689\ \text{M}}{2.76\ \text{M}} = 61.19\% \tag{S10}$$

Comparison of POAs. Comparison of the synthesis methods and the POA, H_2O_2 concentrations in three different POAs is provided in Table S3.

Table S1. Comparison of three POAs.

	POA:H_2O_2 (M:M)	POA conc. (M)	Synthesis Method		
			acid (mL)	H_2O_2 (mL)	reaction time (min)
PFA	100:57	1.971 ± 0.04	2.5	1.5	8
PAA	100:60	4.425	commercial product + additional H_2O_2		
PPA	100:63	1.689 ± 0.029	2	1	15

Text S3. Wastewater sample

The wastewater effluent for this study was obtained from a bench-scale (4 L) mesophilic anaerobic membrane bioreactor (AnMBR) treating synthetic municipal wastewater feed (Table S2). The AnMBR was operated in the sidestream configuration and utilized a hollow-fiber ultrafiltration membrane (membrane surface area ~ 350 cm^2, pore size 30 nm), with a constant, maintained flux of 4.77 LMH at an approximately 24-hour hydraulic retention time (HRT), i.e., flow rate = 2.78 $mL \cdot min^{-1}$ in (4 L/day). Stable operation was maintained over the pH range of 6.8-7.2, an oxidation reduction potential (ORP) of less than -450 mV, and temperature of 34.5-35.5 °C. The anaerobic inoculum was obtained from anaerobic digesters operating at a local wastewater treatment plant in Georgia. Effluents from the AnMBR were collected for the tests of this study on days 42 and 83 of reactor operation, throughout which a constant chemical oxygen demand (COD) removal of >90% was observed. The total nitrogen in the influent was 65.6 ± 10.8 mg $N \cdot L^{-1}$ (n = 26) and the COD in the influent was 592 ± 121 mg $COD \cdot L^{-1}$ (n = 42). Table S3 lists the effluent's wastewater characterization, which was measured by HACH TNT Plus spectrophotometric analyses.

Table S2. AnMBR Synthetic Wastewater Feed Composition

Synthetic Wastewater Feed Component	Concentration ($g \cdot L^{-1}$)
Yeast Extract	0.0525
Skim Milk Powder	0.1118
Sodium Acetate	0.2000
Dextrose	0.2000
Iron DPTA	0.0113
Urea	0.0925
Tryptone	0.0175
Vegetable Oil (in $mL \cdot L^{-1}$)	0.0625
$NaHCO_3$	0.4125
$KHCO_3$	0.3300
NH_4Cl	0.0042
KH_2PO_4	0.0076
$MgHPO_4$*$3H_2O$	0.0190
**Trace Metal Chemical Component**	**Concentration ($mg \cdot L^{-1}$)**
$Cr(NO_3)_3$*$9H_2O$	0.770
$CuCl_2$*$2H_2O$	0.536
$MnSO_4$*$7H_2O$	0.108
$NiSO_4$*$6H_2O$	0.336
$ZnSO_4$	0.208

Table S3. Wastewater Effluent Characterization

Wastewater Parameter	Concentration ($mg \cdot L^{-1}$)
Chemical Oxygen Demand (COD)	35.48 ± 6.04
Total Nitrogen (TN)	67.2 ± 9.55
NH_4^+-N**	43.2 ± 2.6
Total Phosphorus (TP)	10.92 ± 11.14
Alkalinity (as $CaCO_3$)	909.92 ± 96.55

** Two samples used for the disinfection experiments were measured, while the other parameters were measured throughout the AnMBR operation.

Text S4. Bacteria and Virus Measurements

Bacteria Measurement. DifcoTM LB and Difco nutrient agar (VWR International Inc. (Radnor, PA)) were used as non-selective agar for *E. coli* and *Staphylococcus epidermidis*. Eosin methylene blue (EMB) agar (selective for *E. coli*) was obtained from Sigma Aldrich (St. Louis, MO). Mannitol salt agar (selective for *Staphylococcus epidermidis*) was obtained from Research Products International (RPI) (Mt. Prospect, IL). The serially diluted samples were evenly spread on the sterile Petri dishes containing the corresponding agar. The Petri dishes were then incubated at 35 °C for 18 to 24 h. Then, the colony forming unit (CFU) numbers were recorded. Note that both selective and non-selective agars were used for culturing the bacteria, and no obvious difference was found, probably because the bacteria community was dominated by the *E. coli* and *Staphylococcus epidermidis* (10^7 CFU·mL^{-1}).

Virus Measurement (Double Layer Agar Method). Difco^{TM} Tryptic Soy broth (TSB) and agar (TSA) were obtained from VWR International (Radnor, PA). TSA (20 g·L^{-1}) was autoclaved and spread onto petri dishes. Ten g·L^{-1} of TSA was autoclaved and stored at 50-60 °C as the soft agar and used within 3 d. The virus samples were diluted and well-mixed by vortex. For each diluted sample, 4 mL of soft agar, 0.4 mL of host bacteria solution, and 0.1 mL of the sample were gently mixed and decanted to a TSA agar plate. The as-prepared plates were incubated at 35 °C and 25 °C for measurement of MS2 and Φ6, respectively.

Text S5. Genome Damage Measurement

The diluted viruses in phosphate buffer were mixed with the oxidants in the quartz reactors and samples were collected and quenched at defined time intervals. Note that the oxidant concentration applied in the genome degradation tests was higher than that for the infectivity tests. Subsequently, the viral RNA was extracted from the samples with QIAmp viral RNA mini kits (Qiagen, Valencia, CA) according to the manufacturer protocols. Then, 5 μL of extracted RNA samples was mixed with (i) iTaq reaction mixture (Bio-Rad, Hercules, CA), (ii) reverse transcriptase (Qiagen, Valencia, CA), (iii) designed oligonucleotide sequences of primer pairs and Taqman fluorogenic probes for MS2 or Φ6 (Integrated DNA Technologies, Coralville, IA), at proper concentrations, reaching a final volume of 20 μL as manufacturer instructed. Details on target sequences, primers, and probes were provided in the supporting information of Chen et al.'s paper.[3] Finally, the samples were analyzed by one-step quantitative reverse transcription PCR (RT-qPCR) with StepOnePlus real-time PCR (Applied Biosystems, Foster City, CA).[4]

Text S6. Binding Assay

To test the functionality loss of the spike proteins of Φ6, its ability to recognize and attach to its host bacterium *P. syringae* was studied. In order to limit the virus infection at the first step (i.e., inhibiting subsequent genome penetration and replication), the *P. syringae* were either treated in an ice bath[5] or 20 $mg \cdot L^{-1}$ chloramphenicol[6] for 30 min. Then, the Φ6 samples (treated by oxidants) were mixed with the *P. syringae* (1:10 v/v) for 90 min in the ice-bath and room temperature, for the two above-mentioned methods, respectively. Subsequently, the samples were centrifuged and washed for four times by TSB. Each time, we assumed the host bacteria and viruses attached to them should be concentrated in the precipitates, whereas the supernatants containing individual virions should be discarded. Finally, the bacteria precipitate was re-suspended into TSB and the virus genome (attached to the bacteria precipitate) was extracted and measured as described in Text S5.

Despite the non-reactive envelope, the spike proteins of Φ6 (P3) contains cysteine and methionine and could be exposed to POAs outside their envelope. However, the inactivation experiments confirmed that the reactivity of spikes, if at all, did not lead to efficient inactivation of Φ6. As the spike proteins of Φ6 are mainly responsible for the host recognition and attachment,[7] we attempted to test its ability of attachment onto host bacteria after POA treatment. The virus infection of host bacteria contains four stages: (i) attachment (binding onto the bacteria surface), (ii) penetration (incorporation of bacteriophage genome into the host bacteria by endocytosis, injection, or membrane fusion), (iii) replication (amplification of the genome using the enzymes and materials from the host bacteria), (iv) assembly and release.[5,6,8-10] To test the functionality of

host recognition, the infection have to be controlled at the first step. However, we found the previously suggested ice-bath and chloramphenicol methods both could not inhibit the penetration and replication processes, evidenced by the increase of genome concentration over the initial value. We suspect the above mentioned methods may be not applicable for enveloped RNA bacteriophages. For example, it is possible that the ice bath could suppress the injection of genome (MS2 infection pathway[5]), but cannot inhibit the membrane fusion (Φ 6 infection pathway[7]). Mass spectrometry technology might be harnessed to test the structure integrity of the spike proteins, while such effort is beyond the scope of this study. Moreover, it should be note that the damage of spike proteins may not lead to viral inactivation, if the related functionality (i.e., host recognition) is not affected.

Text S7. Chemical Analysis Methods

The concentrations of POAs were measured by the potassium iodide/*N,N*-diethyl-*p*-phenylenediamine (KI-DPD) method with a UV-visible spectrophotometer.[11] In this method, the sample was first dosed with KI and acidified to pH ~ 3 by H_2SO_4, especially for PFA measurements, to keep the POAs protonated and inhibit the reduction of I_3^- by H_2O_2. After DPD addition, the pH was adjusted to > 6 by phosphate buffer (500 mM, pH = 7.0) to enable the reaction between DPD and I_3^-, for which the details were described in our previous study.[11] Although various titration (e.g., permanganate, cerium(IV)) and liquid chromatography (e.g., methyl-*p*-tolyl sulfide (MTS)) methods have been developed for simultaneous quantification of POAs and H_2O_2,[12] these methods were not used to determine POAs in this study due to their relatively long reaction time and the rapid self-decay of PFA.

The total concentration of POA and coexisting H_2O_2 was determined by a horseradish peroxidase-2,2'-azino-bis(3-ethylbenzothiazoline-6-sulfonic)acid (HRP-ABTS) method. The H_2O_2 concentration was obtained by subtracting the POA concentration from the total peroxide concentration. It has been documented that each H_2O_2 oxidizes 2 ABTS to $ABTS^{\bullet+}$ in the presence of HRP, through single electron transfer.[13,14] Herein, we found that a PAA/H_2O_2 mixture oxidizes ABTS to $ABTS^{\bullet+}$ in the presence of HRP, and the total peroxides (PAA and H_2O_2) concentration is always half of the produced $ABTS^{\bullet+}$ (Figure S1), regardless of the PAA/ H_2O_2 molar ratio. In other words, both PAA and H_2O_2 oxidize ABTS (to $ABTS^{\bullet+}$) in the presence of HRP at 1:2 molar ratio. Due to the similarity among POAs, we applied this method for measurement of coexistent H_2O_2 in all of three POA solutions.

The transformation products of cysteine and methionine were studied on LC-HRMS with an RSpak JJ-50 2D column (2.0 ×150 mm, 5 μm).[15] The mobile phase consisted of (A) deionized water with ammonia acetate (50 mM), and (B) methanol: acetonitrile (80:20, v/v). Eluents A and B were run at the ratio of 85:15 with a flow rate at 0.3 $mL \cdot min^{-1}$. The injection volume was 10 μL. The mass spectrometer was set at negative electrospray ionization mode (ESI-) with 140, 180, 220, 250 V fragmentation voltage and 4000 V capillary voltage.

Table S4. Observed Disinfection Rate Constants of Three POAs (k_{obs} in min^{-2})

	E. coli pH 7.1	*E. coli* pH 7.8	*S. epidermidis* pH 7.1	*S. epidermidis* pH 7.8
PFA	2.93	1.03	4.38	3.42
PAA	0.30	0.29	0.63	0.60
PPA	0.28	0.30	0.61	0.63

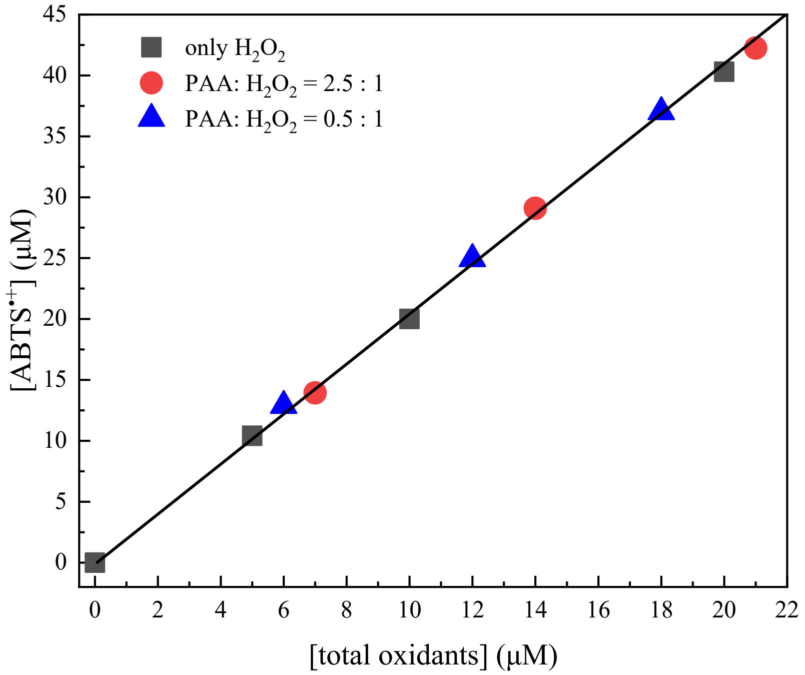

Figure S1. $ABTS^{•+}$ production from PAA and H_2O_2 in the presence of HRP. Experimental conditions: [phosphate buffer] = 10 mM, pH = 6.0, $[ABTS]_0$ = 400 μM.

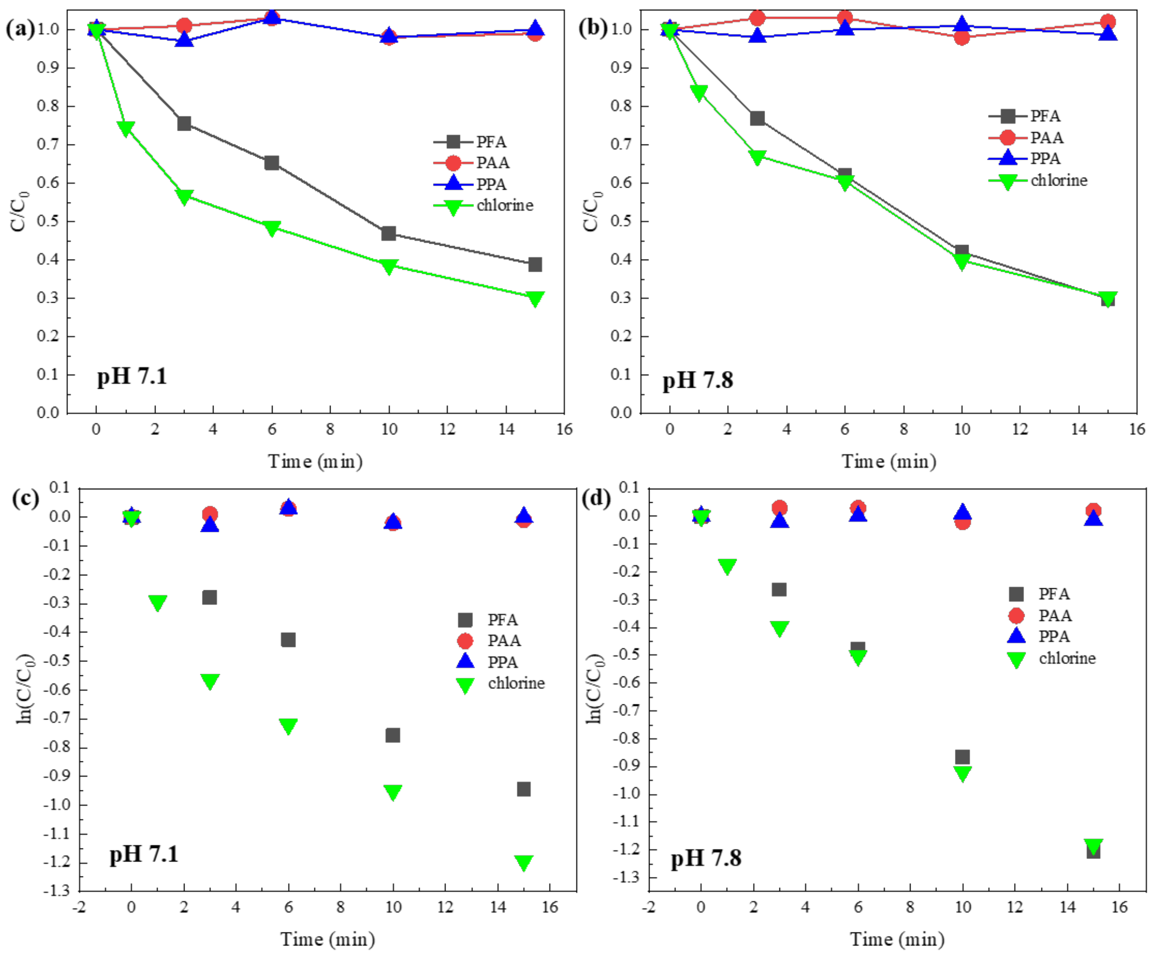

Figure S2. Decay of disinfectants in the effluent from a bench-scale AnMBR. Experimental conditions: [POAs]$_0$ = 120 μM (all with 68-75 μM coexistent H_2O_2), [total chlorine]$_0$ = 120 μM, [phosphate buffer] = 10 mM, temperature = 23 ± 2 °C.

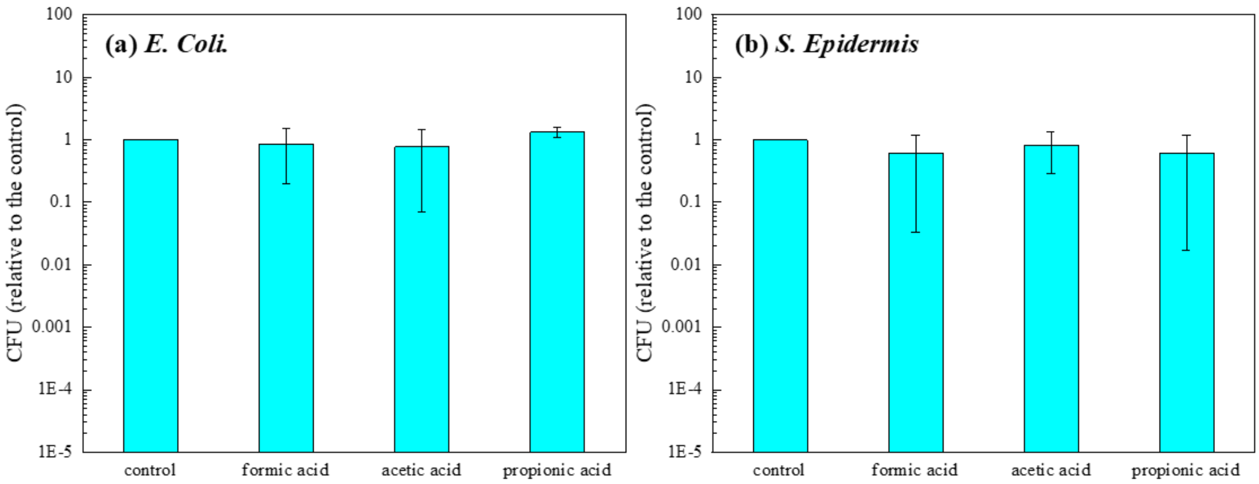

Figure S3. Effects of formic acid, acetic acid, and propionic acid on bacteria growth. Experimental conditions: [carboxylic acids]$_0$ = 1 mM, [phosphate buffer] = 10 mM, pH = 7.1, pretreatment time (before plating) = 5 min.

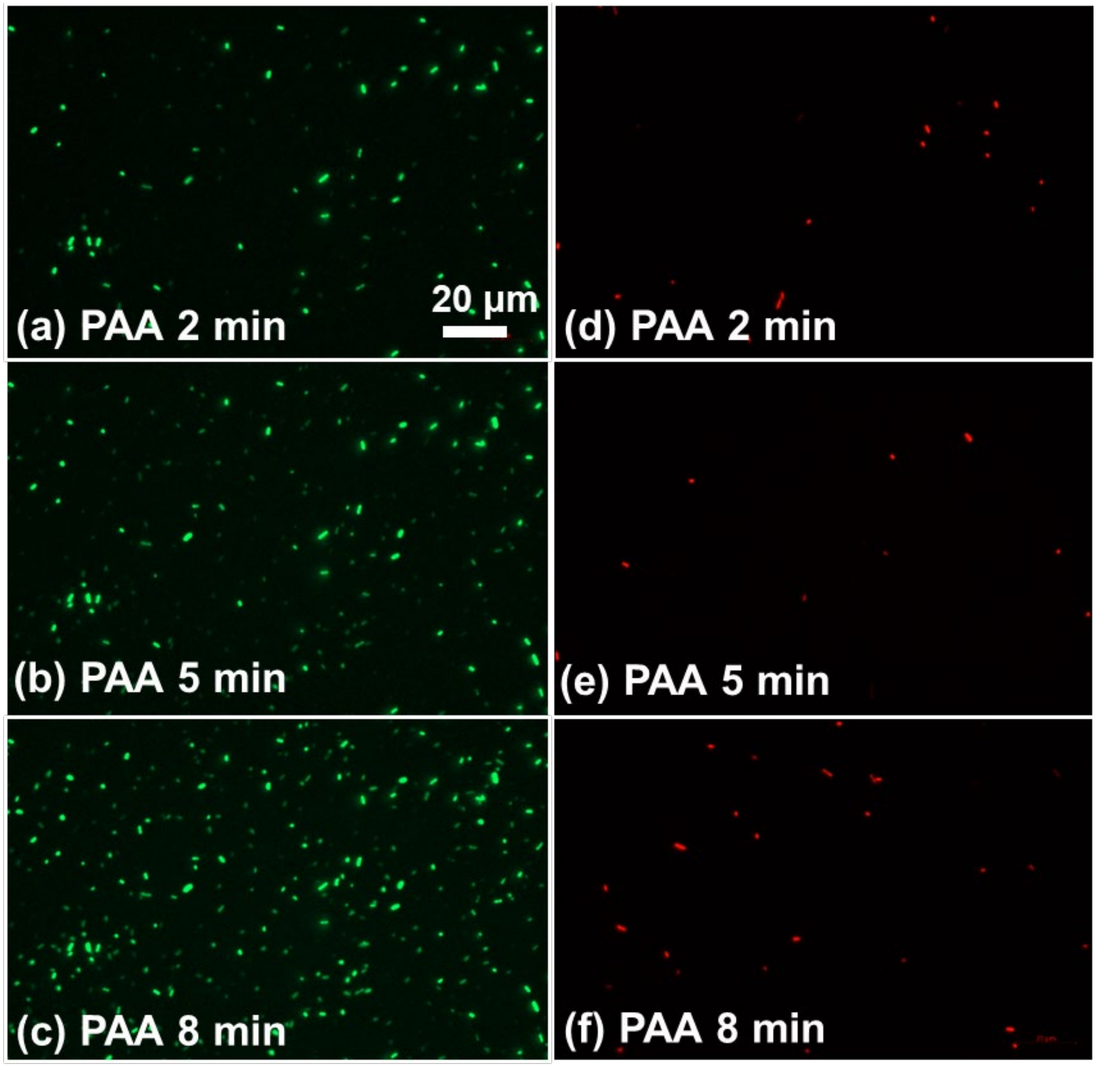

Figure S4. Fluorescence microscope images of DCFH-DA- (a, b, c) or PI- (d, e, f) incubated *E. coli* treated by PAA. Experimental conditions: $[PAA]_0$ = 120 μM (w/ 72 μM of coexistent H_2O_2), $[DCFH\text{-}DA]_0$ = 340 μM, $[PI]_0$ = 15 μM, pH = 7.1, [phosphate buffer] = 10 mM, temperature = 23 ± 2 °C. Notably, DCFH-DA images were taken on the same sample during the oxidation processes, while the PI images were taken on different samples collected and quenched at defined intervals.

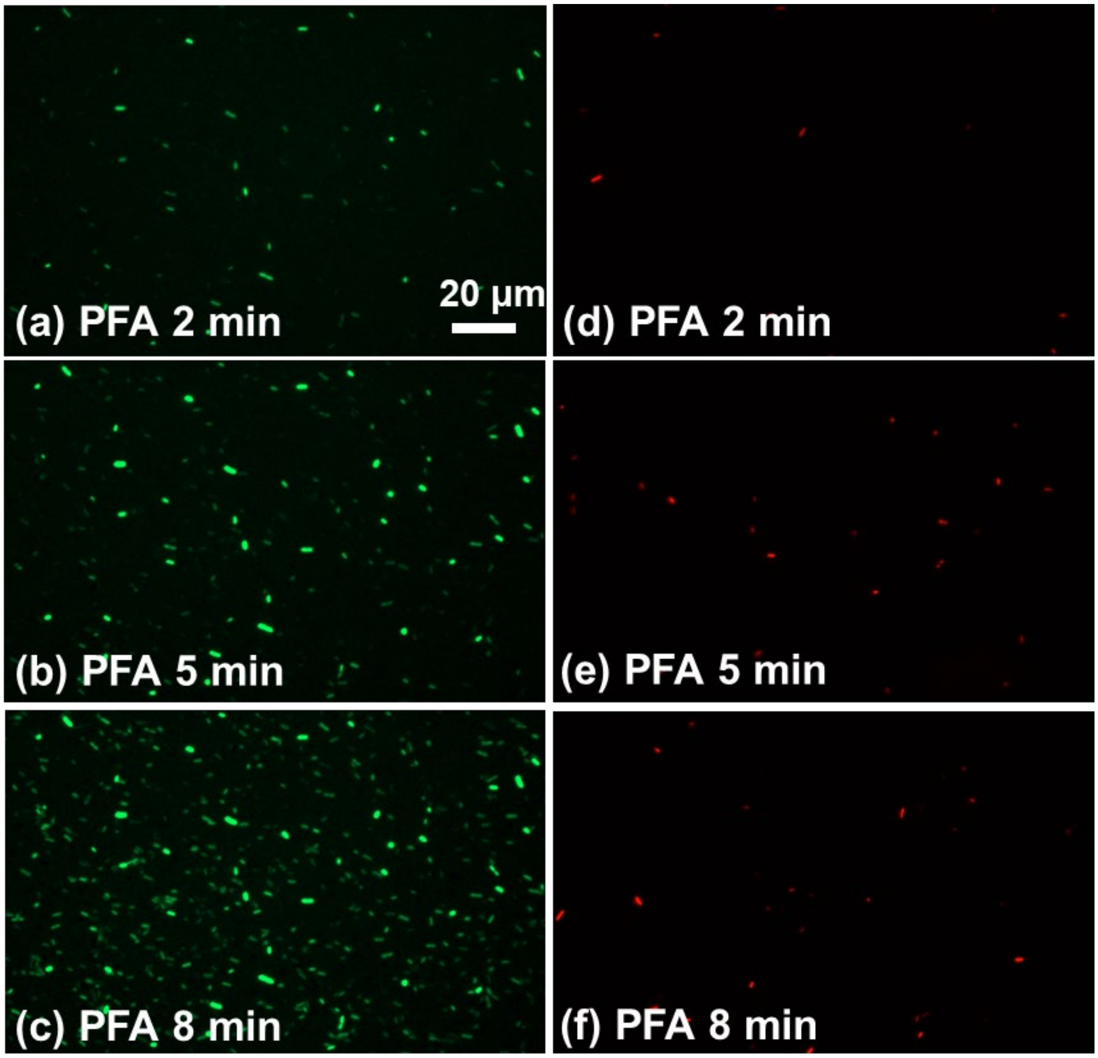

Figure S5. Fluorescence microscope images of DCFH-DA- (a, b, c) or PI- (d, e, f) incubated *E. coli* treated by PFA. Experimental conditions: $[PFA]_0$ = 120 μM (w/ 68 μM of coexistent H_2O_2), $[DCFH\text{-}DA]_0$ = 340 μM, $[PI]_0$ = 15 μM, pH = 7.1, [phosphate buffer] = 10 mM, temperature = 23 ± 2 °C. Notably, DCFH-DA images were taken on the same sample during the oxidation processes, while the PI images were taken on different samples collected and quenched at defined intervals. Thus, the positions of bacteria were consistent throughout the images for the DCFH-DA experiments, but inconsistent for the PI experiments.

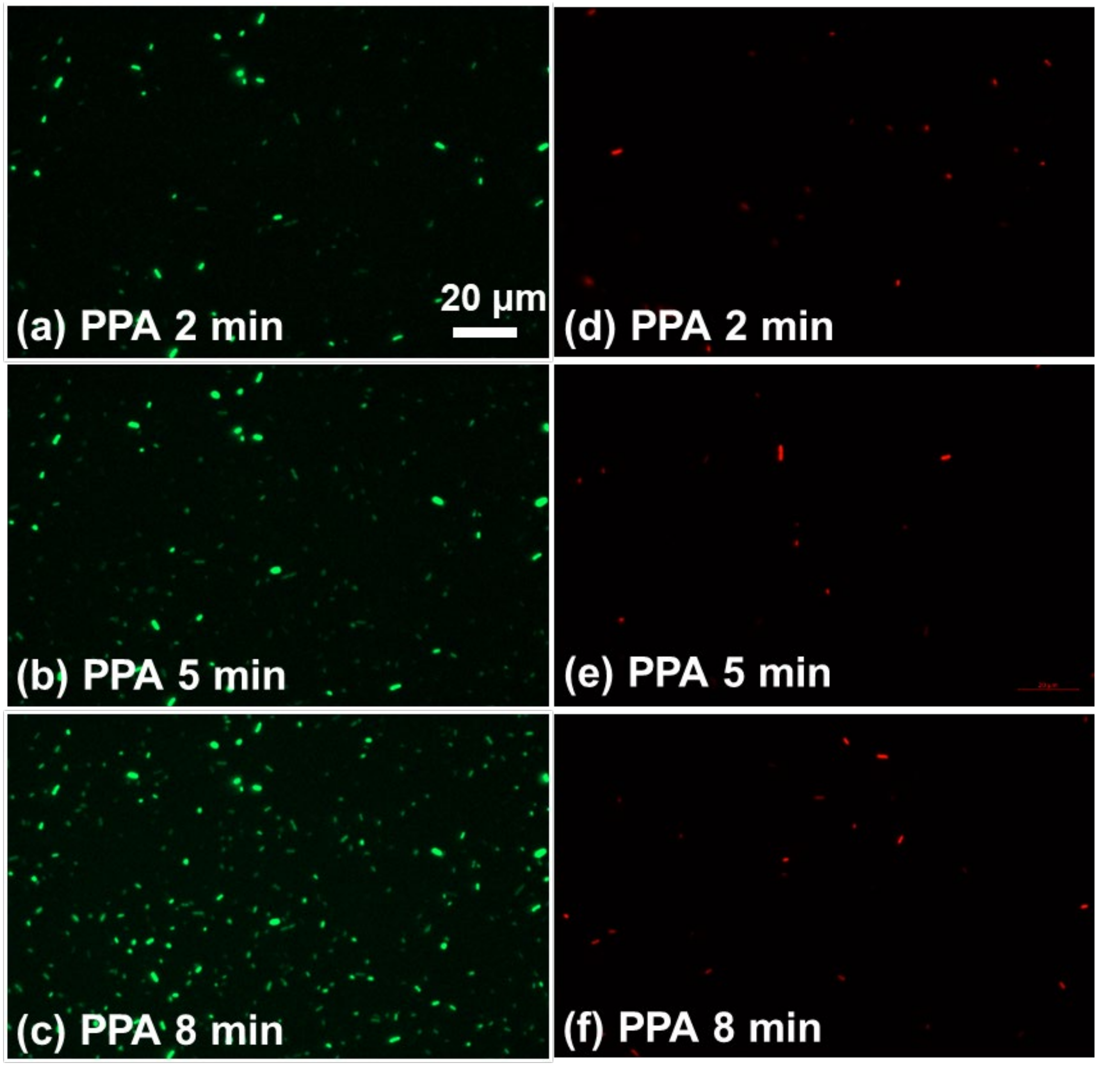

Figure S6. Fluorescence microscope images of DCFH-DA- (a, b, c) or PI- (d, e, f) incubated *E. coli* treated by PPA. Experimental conditions: $[PPA]_0$ = 120 μM (w/ 75 μM of coexistent H_2O_2), $[DCFH\text{-}DA]_0$ = 340 μM, $[PI]_0$ = 15 μM, pH = 7.1, [phosphate buffer] = 10 mM, temperature = 23 ± 2 °C. Notably, DCFH-DA images were taken on the same sample during the oxidation processes, while the PI images were taken on different samples collected and quenched at defined intervals. Thus, the positions of bacteria were consistent throughout the images for the DCFH-DA experiments, but inconsistent for the PI experiments.

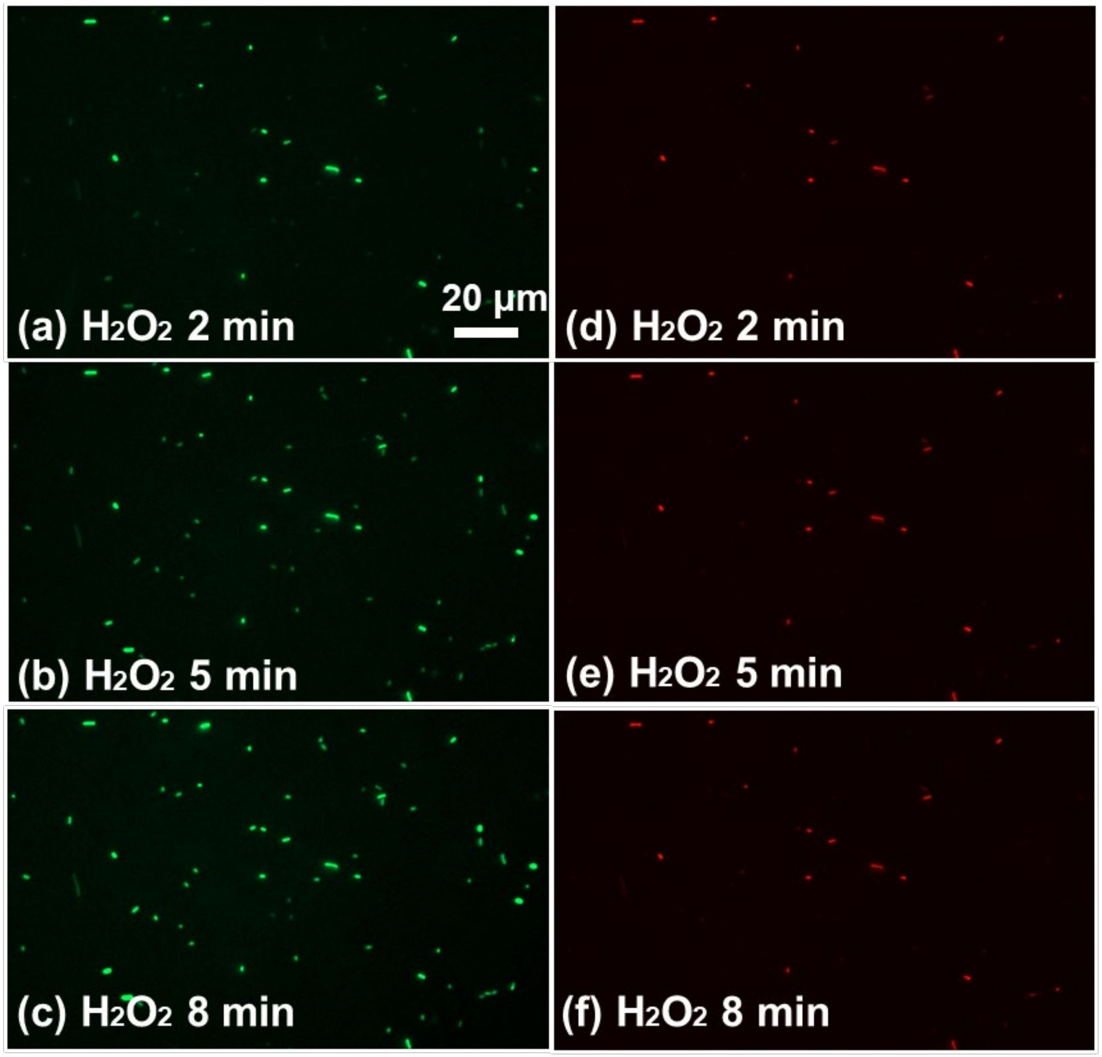

Figure S7. Fluorescence microscope images of DCFH-DA- (a, b, c) or PI- (d, e, f) incubated *E. coli* treated by H_2O_2. Experimental conditions: $[H_2O_2]_0$ = 120 μM, $[DCFH\text{-}DA]_0$ = 340 μM, $[PI]_0$ = 15 μM, pH = 7.1, [phosphate buffer] = 10 mM, temperature = 23 ± 2 °C. Notably, as the reaction between H_2O_2 and iodide is extremely slow, the DCFH-DA and PI images were taken on the same sample during the oxidation processes. Thus, the positions of bacteria were consistent throughout the images for both DCFH-DA and PI experiments, which is different from the POA and chlorine images.

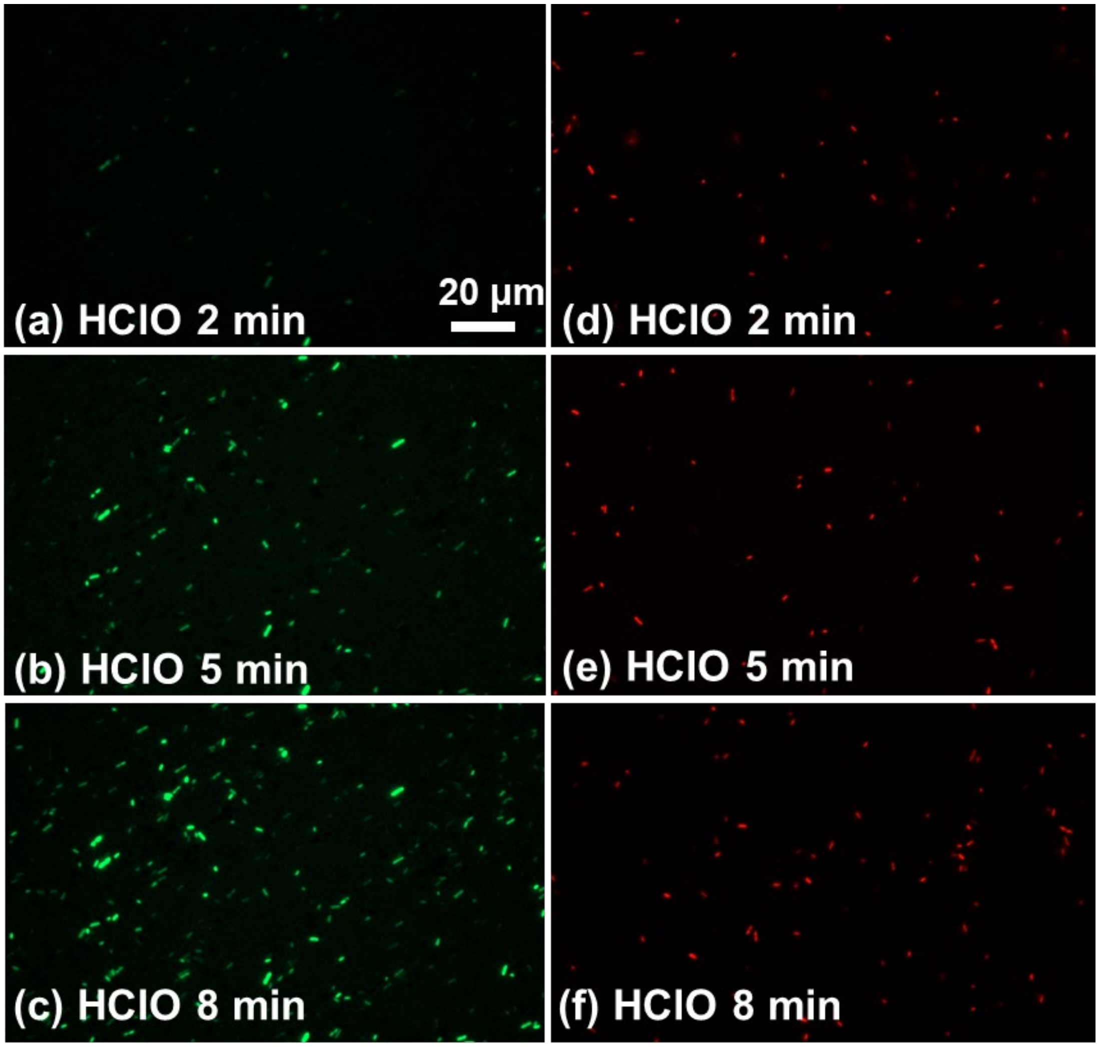

Figure S8. Fluorescence microscope images of DCFH-DA- (a, b, c) or PI- (d, e, f) incubated *E. coli* treated by free chlorine. Experimental conditions: $[\text{free chlorine}]_0$ = 120 μM, $[\text{DCFH-DA}]_0$ = 340 μM, $[\text{PI}]_0$ = 15 μM, pH = 7.1, [phosphate buffer] = 10 mM, temperature = 23 ± 2 °C. Notably, DCFH-DA images were taken on the same sample during the oxidation processes, while the PI images were taken on different samples collected and quenched at defined intervals. Thus, the positions of bacteria were consistent throughout the images for the DCFH-DA experiments, but inconsistent for the PI experiments.

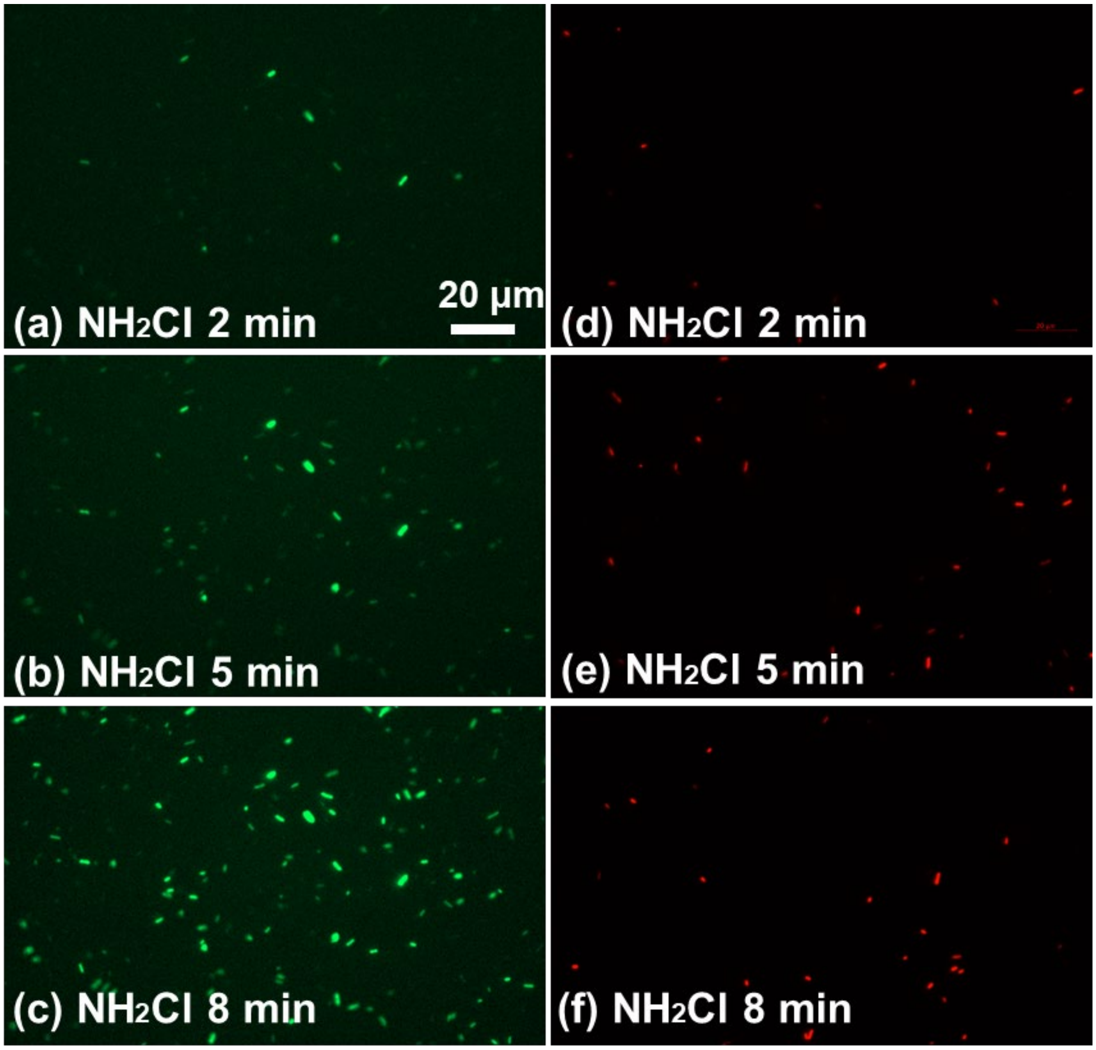

Figure S9. Fluorescence microscope images of DCFH-DA- (a, b, c) or PI- (d, e, f) incubated *E. coli* treated by NH_2Cl. Experimental conditions: $[NH_2Cl]_0$ = 120 μM, $[DCFH\text{-}DA]_0$ = 340 μM, $[PI]_0$ = 15 μM, pH = 7.1, [phosphate buffer] = 10 mM, temperature = 23 ± 2 °C. Notably, DCFH-DA images were taken on the same sample during the oxidation processes, while the PI images were taken on different samples collected and quenched at defined intervals. Thus, the positions of bacteria were consistent throughout the images for the DCFH-DA experiments, but inconsistent for the PI experiments.

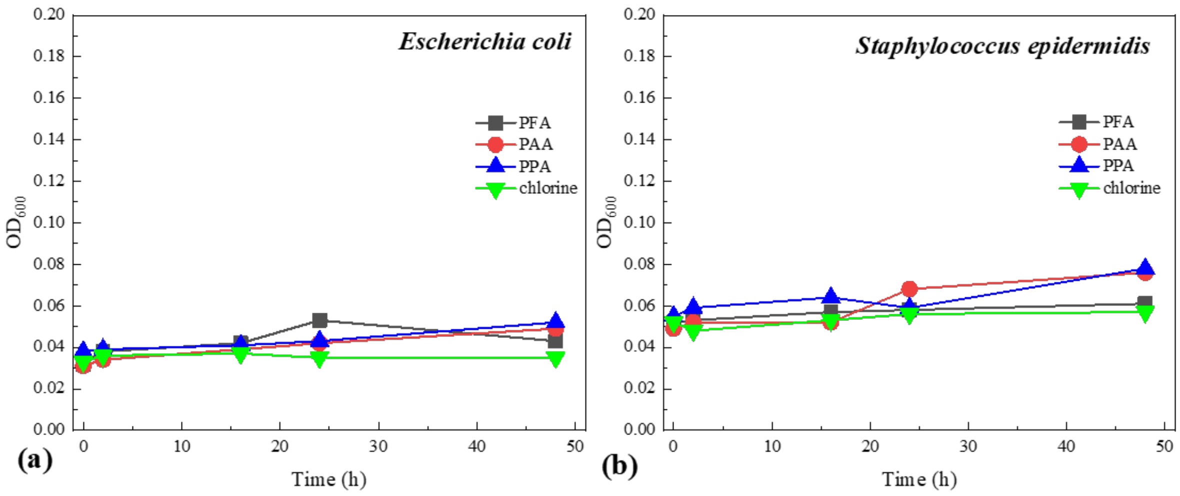

Figure S10. Bacteria regrowth tests (indicated by absorbance at 600 nm) after disinfection by POAs or hypochlorite in AnMBR effluent. Disinfection experimental conditions: $[POAs]_0$ = 120 μM (all with 68-75 μM coexistent H_2O_2), $[\text{total chlorine}]_0$ = 120 μM, $[\text{cells}]_0 \approx 1\times10^7$ CFU·mL^{-1}, pH = 7.1, [phosphate buffer] = 10 mM, disinfection time = 5 min.

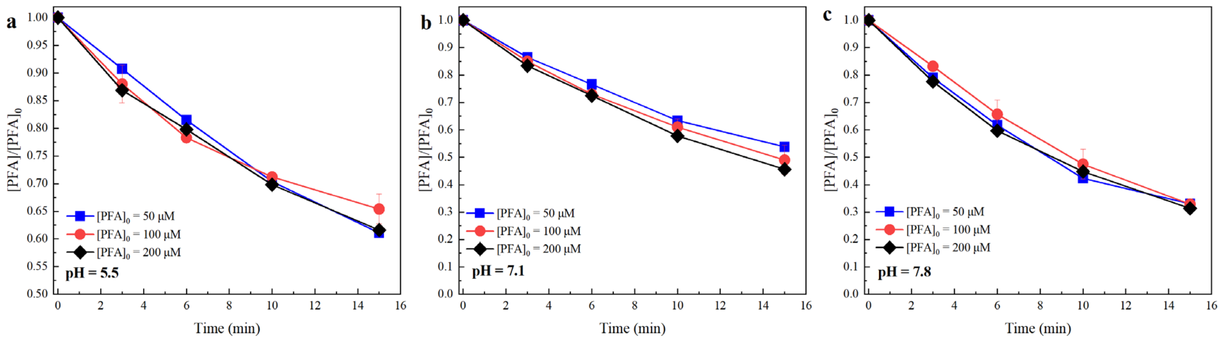

Figure S11. PFA decay pH 5.5 (a), 7.1 (b), and 7.8 (c). Experimental conditions: [phosphate buffer] = 10 mM, temperature = 23 ± 2 °C. Error bars represent standard deviation between parallel experiments.

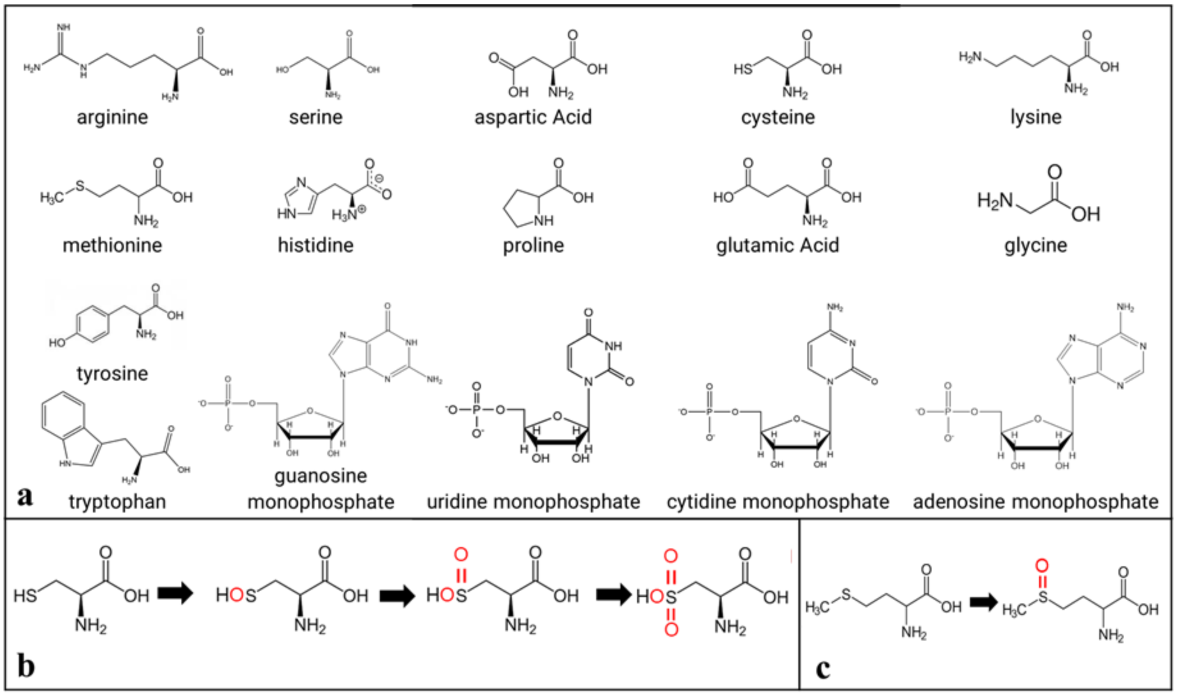

Figure S12. Structures of selected amino acids, ribonucleotides (a), oxidation products of cysteine (b), and methionine (c). Experimental conditions: $[POA]_0 = 20$ mM, $[cysteine]_0 = [methionine]_0 = 10$ mM, pH = 7.1, [phosphate buffer] = 10 mM, reaction time = 30 min, temperature = 23 ± 2 °C. *Note:* Mechanisms in (b) and (c) are based on previous work.[15]

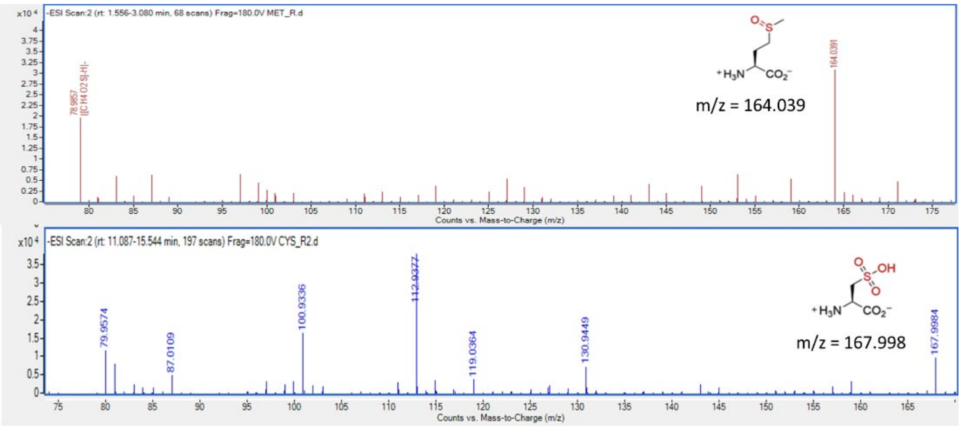

Figure S13. LC-HRMS peaks for the oxidation products of cysteine and methionine. The m/z values are reported for ESI negative mode ions (-H). Experimental conditions: $[POA]_0$ = 20 mM, $[cysteine]_0$ = $[methionine]_0$ = 10 mM, pH = 7.1, [phosphate buffer] = 10 mM, reaction time = 30 min, temperature = 23 ± 2 °C.

References

1. Zhang, T.; Wang, T.; Mejia-Tickner, B.; Kissel, J.; Xie, X.; Huang, C.-H., Inactivation of bacteria by peracetic acid combined with ultraviolet irradiation: Mechanism and optimization. *Environ. Sci. Technol.* **2020,** *54* (15), 9652-9661.
2. Cai, M.; Sun, P.; Zhang, L.; Huang, C.-H., UV/peracetic acid for degradation of pharmaceuticals and reactive species evaluation. *Environ. Sci. Technol.* **2017,** *51* (24), 14217-14224.
3. Chen, W.; Mei, E.; Xie, X., Virus stabilization with enhanced porous superabsorbent polymer (PSAP) beads for diagnostics and surveillance. *ACS ES&T Water* **2022,** 2 (12), 2378-2387.
4. Chen, W.; Wang, T.; Dou, Z.; Xie, X., Self-driven pretreatment and room-temperature storage of water samples for virus detection using enhanced porous superabsorbent polymer beads. *Environ. Sci. Technol.* **2021,** *55* (20), 14059-14068.
5. Wigginton, K. R.; Pecson, B. M.; Sigstam, T.; Bosshard, F.; Kohn, T., Virus inactivation mechanisms: impact of disinfectants on virus function and structural integrity. *Environ. Sci. Technol.* **2012,** *46* (21), 12069-78.
6. Gall, A. M.; Shisler, J. L.; Marinas, B. J., Characterizing bacteriophage PR772 as a potential surrogate for Adenovirus in water disinfection: A comparative analysis of inactivation kinetics and replication cycle inhibition by free chlorine. *Environ. Sci. Technol.* **2016,** *50* (5), 2522-9.
7. Poranen, M. M.; Mantynen, S.; Ictv Report, C., ICTV virus taxonomy profile: Cystoviridae. *J. Gen. Virol.* **2017,** *98* (10), 2423-2424.
8. Vazquez-Bravo, B.; Goncalves, K.; Shisler, J. L.; Marinas, B. J., Adenovirus replication cycle disruption from exposure to polychromatic ultraviolet irradiation. *Environ. Sci. Technol.* **2018,** *52* (6), 3652-3659.
9. Gall, A. M.; Shisler, J. L.; Marinas, B. J., Analysis of the viral replication cycle of Adenovirus serotype 2 after inactivation by free chlorine. *Environ. Sci. Technol.* **2015,** *49* (7), 4584-90.
10. Gall, A. M.; Shisler, J. L.; Mariñas, B. J., Inactivation kinetics and replication cycle inhibition of Adenovirus by monochloramine. *Environ. Sci. Technol. Lett.* **2016,** *3* (4), 185-189.
11. Wang, J.; Kim, J.; Ashley, D. C.; Sharma, V. K.; Huang, C.-H., Peracetic acid enhances micropollutant degradation by ferrate(VI) through promotion of electron transfer efficiency. *Environ. Sci. Technol.* **2022,** *56* (16), 11683-11693.
12. Kim, J.; Huang, C.-H., Reactivity of peracetic acid with organic compounds: A critical review. *ACS ES&T Water* **2020,** *1* (1), 15-33.
13. Zhang, T.; Huang, C.-H., Modeling the kinetics of UV/peracetic acid advanced oxidation process. *Environ. Sci. Technol.* **2020,** *54* (12), 7579-7590.
14. Zhu, J.; Yu, F.; Meng, J.; Shao, B.; Dong, H.; Chu, W.; Cao, T.; Wei, G.; Wang, H.; Guan, X., Overlooked role of Fe(IV) and Fe(V) in organic contaminant oxidation by Fe(VI). *Environ. Sci. Technol.* **2020,** *54* (15), 9702-9710.
15. Du, P.; Liu, W.; Cao, H.; Zhao, H.; Huang, C.-H., Oxidation of amino acids by peracetic acid: Reaction kinetics, pathways and theoretical calculations. *Water Res. X* **2018,** *1*, 100002.
